# Does Learning Influence the Detection of Signals in a Response-Inhibition Task?

**DOI:** 10.5334/joc.73

**Published:** 2019-07-31

**Authors:** Maisy Best, Frederick Verbruggen

**Affiliations:** 1 University of Glasgow, UK; 2 Ghent University, BE

**Keywords:** Cognitive Control, Attention, Executive functions, Learning

## Abstract

Learning can modulate various forms of action control, including response inhibition. People may learn associations between specific stimuli and the acts of going or stopping, influencing task performance. The present study tested whether people also learn associations between specific stimuli and features of the stop or no-go signal used in the task. Across two experiments, participants performed a response-inhibition task in which the contingencies between specific stimuli and the spatial locations of the ‘go’ and ‘withhold’ signals were manipulated. The contingencies between specific stimuli and either going or withholding were also manipulated, such that a subset of stimuli were associated with responding and another subset with withholding a response. Although there was clear evidence that participants learned to associate specific stimuli with the acts of going or withholding, there was no evidence that participants acquired the spatial signal-location associations. The absence of signal learning was supported by Bayesian analyses. These findings challenge our previous proposals that learning always influences signal-detection processes in response-inhibition tasks where features of the signal remain the same throughout the task.

The ability to stop irrelevant actions is considered to be a core executive control function and has been linked to behavioural outcomes in healthy and clinical populations (e.g. Chambers, Garavan, & Bellgrove, 2009; Miyake et al., 2000; Ridderinkhof, van den Wildenberg, Segalowitz, & Carter, 2004; Verbruggen & Logan, 2008a). Although executive control has been the subject of extensive research over the past century, many theories still attribute executive control to an ill-defined set of ‘executive’ control functions without explaining how control is achieved (Verbruggen, McLaren, & Chambers, 2014). This is problematic because the outcomes of executive control (e.g. to inhibit a prepotent response) and the tasks used to measure control (e.g. stop-signal task) are too readily confused with the mechanisms through which control is achieved (e.g. ‘response inhibition’) meaning that the basic cognitive processes that contribute to successful stopping are often ignored. To address this, Verbruggen, McLaren, et al. (2014) proposed a theoretical framework for action control. Inspired by the seminal work of Sternberg (1969) and others, they argued that most forms of action control also depend on three basic cognitive processes (signal detection, action selection, and action execution), which are modulated via correction- or evaluation mechanisms, proactive strategy adjustments, task rules maintained in memory, and learning (Verbruggen, McLaren, et al., 2014). The aim of this framework is to eliminate the control ‘homunculi’ from theories of action control. In the present study, we tested part of this framework by examining how learning modulates signal detection in a response-inhibition task.

One of the first stages of inhibiting a response often involves detecting an external stop signal (e.g. noticing an oncoming vehicle when crossing a road). Computational modelling work suggests that a considerable proportion of the stopping latency is occupied by perceptual or afferent processes (Boucher, Palmeri, et al., 2007; Logan, Van Zandt, et al., 2014; Salinas & Stanford, 2013), and behavioural studies demonstrate that stopping performance is impaired when perceptual distractors are presented (e.g. Verbruggen, Stevens, & Chambers, 2014). Electrophysiology studies have also linked intra- and inter-individual differences in stopping performance with signal-detection processes (e.g. Bekker et al., 2005; Elchlepp & Verbruggen, 2017). Early sensory event-related potential components (such as the N1 and the Selection Negativity), for example, are modulated in contexts where participants are instructed to monitor for a stop signal but not in contexts where participants are instructed to ignore the stop signals (Elchlepp, Lavric, et al., 2016; Hong, Wang, Sun, Li, & Tong, 2017; Langford, Krebs, Talsma, Woldorff, & Boehler, 2016). These findings suggest that people readily adjust their attentional settings in situations where they are required to monitor for a stop signal. Combined, these findings indicate that signal detection seems to play a critical role in performance of response-inhibition paradigms, such as the stop-signal task.

Previous research has shown that the implementation of cognitive control in various tasks or situations can be modulated by acquired associations between stimuli, responses, tasks, or even control settings (for an overview, see Egner, 2014). In the present study we tested if signal-detection processes could also be modulated by learning in response-inhibition tasks. Several previous studies have demonstrated that inhibitory control can be triggered via the episodic retrieval of stimulus-stop associations from memory (e.g. Best, Lawrence, Logan, McLaren, & Verbruggen, 2016; Bowditch, Verbruggen & McLaren, 2016; Chiu, Aron, & Verbruggen, 2012; Liefooghe, Degryse, Theeuwes, 2016; Verbruggen, Best, et al., 2014; Verbruggen & Logan, 2008b). We proposed that there are at least two pathways through which learning in response-inhibition tasks could influence behaviour (Verbruggen, Best, et al., 2014; for a simplified version of this associative architecture, see Figure 1). First, there is the *direct* pathway in which a link between a stimulus and the stop goal is formed (solid lines, Figure 1). The direct pathway was assumed in Verbruggen and Logan’s (2008b) original ‘automatic inhibition’ hypothesis. It is via the direct pathway that stimulus-stop associations are formed. The retrieval of these associations can, at least partly, activate the stop goal, which slows responding when participants are required to respond to stop-associated stimuli and improves inhibition when participants are required to stop (i.e. both go and stop performance are influenced by the direct route). Second, there is the *indirect* pathway in which a link is formed between a stimulus and a (perceptual) representation of whatever stop signal was used in the task (dashed lines, Figure 1). The *indirect* pathway exploits the consistent association between the go stimulus and the stop signal in typical response-inhibition tasks. Under these circumstances, the presentation of a stop-associated stimulus would facilitate stop-signal detection rather than activate a representation of the stop goal or the stop response (as originally proposed by Verbruggen & Logan, 2008b). Thus, learning via the indirect pathway would influence performance on stop trials (when the stop signal is presented) but would not (or at least to a smaller extent) influence performance on go trials (when the stop signal is not presented). Behaviourally, this would manifest as a learning effect on the probability of stopping but not on go reaction times (i.e. stop but not go performance is influenced by learning).

**Figure 1:**
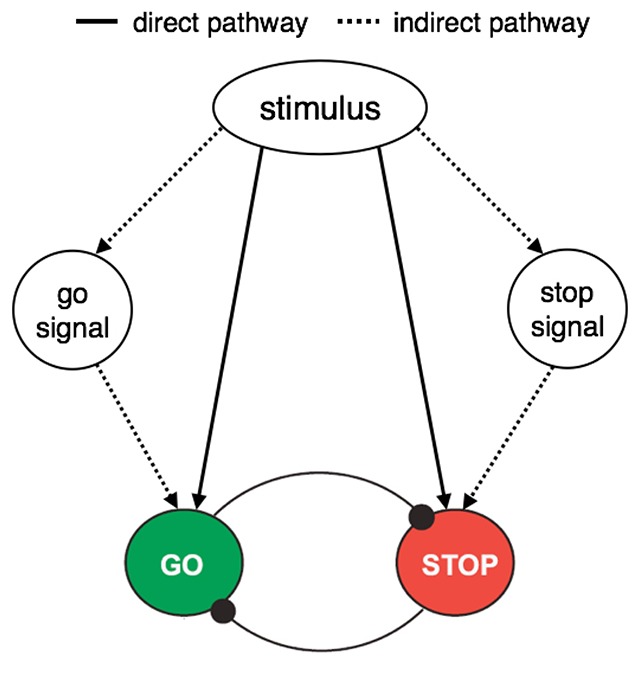
Overview of the architecture of the associative stop system (for a more detailed overview, see Verbruggen, Best, et al., 2014). There are two associative routes to activating the stop-goal; a direct pathway between the stimulus and the go/stop goal (solid lines) and an indirect pathway between the stimulus or cue and the go/stop goal that is mediated via a representation of the go/stop signal (dashed lines).

In support of the indirect pathway, research showed that slowing on go trials was more pronounced in a condition where the stop-associated stimuli were paired with multiple coloured stop signals (weakening the stimulus-signal link) compared with a condition where the stop-associated stimuli were paired with a single coloured stop signal (Bowditch, Verbruggen, et al., 2016). This suggests that participants in the ‘multiple signal’ group acquired more direct associations between the stop-associated stimuli and the stop goal than participants in the ‘single signal’ group.

In the present study, we focused on a different perceptual feature of the stop signal, namely its spatial location. We focused on spatial attention for two main reasons. First, in the original Verbruggen, Best, et al. (2014) study in which we proposed the indirect route hypothesis, the visual stop signal always appeared below the the go stimulus. Thus, subjects had to pay attention to visual features that appeared at different locations. Attention in this task might have been modulated by learning. For example, Chun and Jiang (1998) showed that detection of visual targets (amongst distractors) was enhanced when the target always appeared in the same spatial configuration (‘context’). Binding of various stimulus features or attributes (including colours and spatial locations) has also been observed in other paradigms or situations, improving performance when the features match, but impairing performance when there is a mismatch (see e.g. Park & Kanwisher, 1994). Thus, we speculated that stop-signal detection could be similarly enhanced if it always (or most often) appeared in the same location with the same go stimulus. Second, signal-detection processes and visual attention play a critical role in response-inhibition tasks when stop signals and go stimuli appear at different locations. For example, in the above-mentioned study by Verbruggen, Stevens, et al. (2014), perceptual distractors primarily influenced stopping when the signals occurred in the periphery. In another study, Elchlepp and Verbruggen (2017) used lateralised ‘withhold’ signals (the letters ‘M’ and ‘W’) in a variant of the stop-change paradigm. In that study, the presentation of the lateralised signals elicited early perceptual event-related potential components (N1pc, P2pc, N2pc) that index spatial shifts of attention, indicating that participants shifted their attention to the periphery in order to facilitate signal detection. Furthermore, the peaks of these early perceptual components were numerically delayed on slow compared to fast change trials, indicating that fast responding to lateralised withhold/change signals depends on the allocation of attentional resources to the spatial location of the signal. However, Elchlepp and Verbruggen (2017) did not examine how learning influenced signal detection in their response-inhibition task. As stop signals often occur in the periphery in real life (e.g. detecting pedestrians crossing the street when driving), we argue that it is also important to examine how learning influences the detection of such spatial stop (and go) signals.

In the present study, we used an adapted version of the Elchlepp and Verbruggen (2017, Exp. 1) task (which induced measurable shifts of spatial attention) to investigate whether learning influences the detection of lateralised go and withhold signals. On each trial in our task, participants were presented with an arbitrary word cue in the centre of the screen that preceded the presentation of a withhold or go signal to the right or to the left of the cue, and a visual distractor on the opposite side^1^. As in the Elchlepp and Verbruggen (2017) task, our paradigm shared features of stop-signal and go/no-go tasks so we use the term ‘withhold’ instead of ‘stop’ or ‘no-go’. Note that Elchlepp and Verbruggen found that the amplitudes of the N2 and P3 event-related potentials were greater on withhold trials than on go trials. These components are often present on stop and no-go trials in typical stop-signal and go/no-go tasks, and are interpreted as reflecting processes involved in successful response inhibition (e.g. Wessel & Aron, 2015). Since our task is a variant of the Elchlepp and Verbruggen (2017) task, performance on withhold trials in the present task likely relies on similar inhibitory control processes as in typical stop-signal and go/no-go tasks used in the response-inhibition literature.

To examine stimulus-signal learning in our task, the contingencies between the cues and withhold (or go), and the contingencies between the cues and the spatial location of the withhold (or go) signal were manipulated (unbeknown to participants). It follows from our earlier ‘indirect’ pathway hypothesis that the acquisition of the associations between the cues and the spatial-signal location would result in improvements to task performance for cues consistently paired with the same signal location (and hence, a consistent ‘context’, see e.g. Chun & Jiang 1998) compared with cues paired equally with both signal locations (Experiment 1). These improvements would reflect the facilitation of task performance by training-induced improvements in signal detection. Furthermore, performance on trials in which the signal appeared in the trained location would be better than on trials in which the signal appeared in the untrained location (Experiment 2). Evidence that participants acquired the associations between the cues and the signal location would provide the strongest evidence to date for the idea that learning influences signal detection in response-inhibition tasks.

## Experiment 1

In Experiment 1, we varied the spatial location of coloured go/withhold signals on the screen. On each trial, for a subset of cues, the relevant signals were consistently presented in the same signal locations; for another subset, the signals were presented in two signal locations with equal probability. In line with our earlier proposal, we hypothesised that if participants learn the spatial cue-signal associations, responding on go trials would be faster and the probability of responding on withhold trials would be lower for cues presented with go or withhold signals in the same spatial location (or in other words, when the overall ‘context’ was consistent) than for cues presented with go or withhold signals in two locations with equal probability.

A subset of cues was also consistently associated with withhold and another subset was consistently associated with go. This experiment was designed as a pilot for an electrophysiology experiment so, to maximise the available trials, the withhold-associated cues were paired with ‘withhold’ and the go-associated cues with ‘go’ on 100% of presentations. Thus, it was not possible to directly compare task performance for the withhold-associated and go-associated cues. However, previous work shows that the acquisition of the stimulus-withhold associations is reflected in expectancies following task completion (Best et al., 2016). Therefore, immediately after completing the task participants were asked to rate the extent to which they expected to withhold their response for each of the cues presented in the task. We also examined whether these expectancies correlated with overall improvements in task performance which likely reflect a combination of item-specific learning and general task learning.

We obtained four measures of task performance. To index inhibitory control, we measured the probability of responding on stop (withhold) trials [*p*(respond|signal)] (note that this experiment was not designed to estimate the covert latency of the stop process). Performance in response-inhibition paradigms is typically described in terms of a race between two competing processes: a *go* process and a *stop* process (Logan, 1981; Logan & Cowan, 1984). According to this race model, the probability of responding on a stop trial depends on the relative finishing times of the go and stop processes. When the stop process finishes before the go process, response inhibition is successful and the response is successfully withheld; when the go process finishes before the stop process, response inhibition is unsuccessful and a response is incorrectly emitted. Consistent with the response-inhibition literature, we also obtained three measures of performance on go trials: reaction times, the probability of a missed response, and the probability of a correct choice response. Go reaction times index the latency of the go process which should decrease with task practice. Proactive response-strategy adjustments and stimulus-signal learning could result in a higher percentage of omitted responses as well as changes in accuracy, so we distinguished between the probability of a missed go response [*p*(miss)] and probability of a correct go response [*p*(correct)]: *p*(miss) is the ratio of the number of missed go responses to the total number of go trials, and *p*(correct) is the ratio of the number of correct go responses to the number of go responses with a response (see also e.g. Verbruggen & Logan, 2009).

### Method

#### Participants

Thirty-six volunteers from University of Exeter participated for monetary compensation (£5) or partial course credit (*M* = 20.39 years, *SD* = 3.10, 30 females, 33 right-handed). Five participants were excluded as they had less than 70% correct go responses. All experiments were approved by the local research ethics committee at the School of Psychology, University of Exeter (approval number 2015/931). Written informed consent was obtained after the nature and possible consequences of the study were explained. The target sample size and exclusion criteria were decided in advance of data collection. We conducted a power calculation using G*Power 3.1 (Faul, Erdfelder, Lang, & Buchner, 2009) to ensure that we had enough power (0.80) to detect medium-sized effects (Cohen’s *d* = 0.5) in the (one-tailed) within-subject comparisons. Note that we based our power calculation on one-tailed comparisons because the ‘indirect signal-learning’ account makes strong predictions about the direction of the RT and *p*(respond|signal) effects. No differences between cue types or differences in the opposite direction (i.e. shorter RTs or lower *p*(respond|signal) for inconsistent cues (paired with signals on the left and right) than for consistent cues (paired with signals in the same location) would argue against the indirect signal learning pathway.

#### Apparatus, stimuli, and procedure

**Figure 2:**
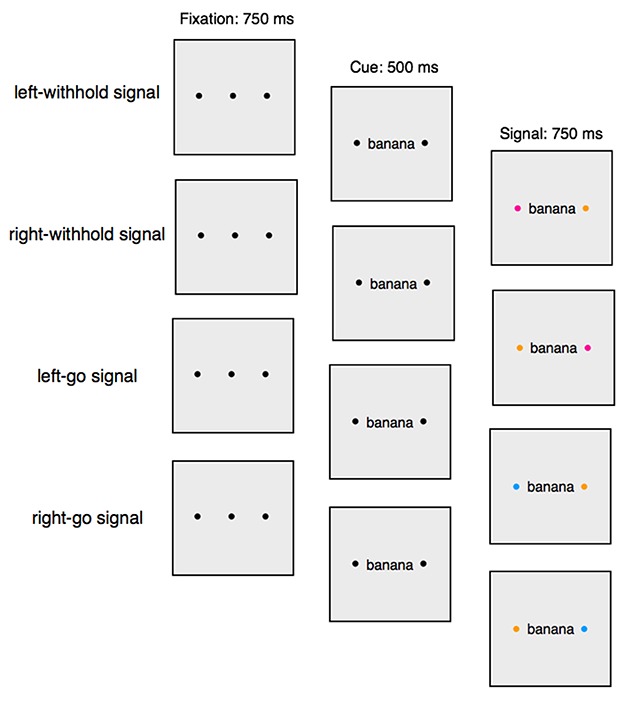
The left-withhold signal, right-withhold signal, left-go signal, and right go signal trial structures in Experiment 1. In this example the withhold signal is represented as a pink dot, the go signal is represented by a blue dot and the distractor is represented by a yellow dot. The dot colours were fully counter balanced across participants.

The experiment was run on a 21-inch iMac (screen size: 1920 × 1080 pixels) using Psychtoolbox (Brainard, 1997). The cues were words presented in black lowercase font (Courier 16 point) and the go and withhold signals were coloured dots (size: 20 × 20 pixels) presented on a grey background (RGB: 255 255 255; Figure 2). The word was presented in the centre of the screen and a coloured go or withhold signal to the left or to the right of the word (word-signal distance: 160 pixels or 2 cm). A distractor (in a different colour) was presented on the opposite side. We created a list of 112 five- and six-letter words (for full wordlists, see Supplementary Material). The word could refer to a natural or a human-made object (56 natural words, 56 human-made words).

The experiment comprised three parts, with four blocks per part. Each word was presented once per block. As shown in Table 1, there were six different cue-types. Words were randomised over the cue-types across participants. Note that there were no ‘withhold/go-inconsistent but location-consistent’ cues.

**Table 1:** The proportion of left withhold signal, right withhold signal, left go signal, and right go signal trials as a function of withhold/go-type in Experiment 1. Note that for the analyses, the left-withhold and right-withhold cues were averaged (*withhold-consistent* cues) and the left-go and the right-go cues were averaged (*go-consistent* cues) as these cues consistently predicted the presentation of withhold/go signals in a single location. The *withhold-inconsistent* and *go-inconsistent* cues predicted the presentation of withhold/go signals in two locations (to the left and to the right) with equal probability.

Cue-type	No. of words	% left withhold signal	% right withhold signal	% left go signal	% right go signal

**Withhold-left**	12	100	0	0	0
**Withhold-right**	12	0	100	0	0
**Withhold-inconsistent**	24	50	50	0	0
**Go-left**	16	0	0	100	0
**Go-right**	16	0	0	0	100
**Go-inconsistent**	32	0	0	50	50

*Note*: The overall *p*(withhold-signal) was 0.43.

On each trial, three black fixation dots were presented, marking the central, left and right stimulus locations (Figure 2). After 750 ms, the central fixation dot was replaced with a word. After 500 ms, the fixation dots to the left and to the right of the word were replaced with coloured dots (aside from the colour change, all other features of the dots remained the same as the fixation dots). There were three dot colours: yellow (RGB: 255 155 0), pink (RGB: 255 0 155), and blue (RGB: 0 155 255). On go trials, one of the dots to the left or to the right of the word was presented in the go-signal colour (e.g. left = blue) and the dot on the other side was presented in the distractor colour (e.g. right = yellow). On go trials, participants had to decide whether the word referred to a natural or human-made object. Participants were told that they “should respond as quickly and as accurately as possible on these trials” via on-screen instructions (for full instructions, see Supplementary Materials). Half of the participants had to press the ‘c’ key (with their left index finger) when the word referred to a natural object and the ‘m’ key (with their right index finger) when the word referred to a human-made object. This mapping was reversed for the other half of participants. The word, signal, and the distractor remained on the screen for 750 ms (hence, the maximum RT was 1250 ms). On withhold trials, one of the dots to the left or to the right of the word was presented in the withhold-signal colour (e.g. left = pink) and the dot on the other side of the word was presented in the distractor colour (e.g. right = yellow). The distractor was the same on go and withhold trials. The go, withhold, and distractor colours remained the same throughout the experiment, but were counterbalanced across participants (e.g. P1: blue = go, pink = withhold, yellow = distractor; P2: yellow = go, blue = withhold, pink = distractor). To increase the likelihood that participants would be required to inhibit a planned response on withhold trials, participants were instructed to begin preparing their go response following the presentation of the word (i.e. the cue; as in Elchlepp & Verbruggen, 2017). At the end of each block, participants received feedback on their mean RT on go trials, the number of go errors, the number of missed go responses, and the percentage of failed stops during that block (i.e. our four main dependent variables; see above). After 15 seconds, participants had to press any key to start the next block.

Following completion of the experimental task, each word was again presented on the screen (order randomised anew for each participant). Participants were asked to rate “How much do you expect to withhold your response when this word is presented?” on a 9-point scale (1 = *I definitely do not think this word indicates that I have to withhold my response;* 9 = *I definitely think this word indicates that I have to withhold my response*). There was no response deadline for the ratings.

#### Analyses

All data processing and analyses were completed using R (R Development Core Team, 2014). All data files and R scripts are deposited in the Open Science Framework (https://osf.io/j6tk9/?view_only=329b1312f7524ef58bb43050e5100cd0).

For all analyses, we collapsed the *withhold-left* and *withhold-right* cues as both of these cues consistently predicted a given signal location (herein *withhold-consistent* cues). For the same reason, we also collapsed the *go-left* and *go-right* cues (herein *go-consistent* cues). Note that the delay between the presentation of the cues and the signals was always 500 ms so we could not estimate or analyse stop-signal reaction time. Consistent with previous studies, we analysed the probability of responding on withhold trials [*p*(respond|signal)] to determine if withhold learning influenced stopping performance (see also e.g. Best et al., 2016; Bowditch, Verbruggen, et al., 2016; Noël, Brevers, et al., 2016; Verbruggen, Best, et al., 2014; Verbruggen & Logan, 2008b).

ANOVAs were performed on correct RTs, the probability of correct responses, and the probability of missed responses on go trials, and on the probability of responding on withhold trials. To examine learning, behavioural performance was analysed as a function of ‘part’ (there were 4 blocks per part: 1 = blocks 1–4; 2 = blocks 5–8; 3 = blocks 9–12) and cue-type (withhold-consistent, withhold-inconsistent, go-consistent, go-inconsistent) which were included as within-subjects factors. An ANOVA was also performed on the expectancy ratings with withhold/go-type (withhold-associated cues, go-associated cues) as a within-subjects factor. Where appropriate, we applied the Huyhn-Feldt correction for violations of sphericity. For pairwise comparisons, Hedge’s g_av_ is the reported effect size measure (Lakens, 2013).

To examine support for the null hypothesis, we also computed Bayesian t-tests for all pairwise comparisons and Bayesian regressions for the expectancy-behaviour correlations (Rouder, Morey, Speckman, & Province, 2012). Several methods exist to calculate Bayes factors. The Bayes factors (BF_10_) reported in this paper compare the likelihood of the data under the null hypothesis of no difference against the alternative hypothesis of a difference with an effect size that corresponds to the prior. The default prior (0.707) assumes a medium-to-large difference between conditions. A Bayes factor less than 0.33 constitutes moderate support for the null hypothesis whereas a Bayes factor of more than 3 constitutes moderate support for the alternative hypothesis (for interpretations of Bayes factors, see Schönbrodt & Wagenmakers, 2017). We calculated the Bayes factors with the BayesFactor package in R, using the default prior (Morey, Rouder, & Jamil, 2015).

### Results

#### Go analyses

Go reaction times decreased with training, as reflected in a reliable main effect of part (Table 2). Contrary to our ‘indirect signal-learning’ hypothesis, there was no reliable difference between the go-consistent and go-inconsistent cues and the corresponding Bayes factor provided anecdotal support for the null hypothesis of no difference (*BF_10_* = 0.67). The two-way interaction between part and cue-type was also not reliable.

**Table 2:** Overview of repeated measures Analyses of Variance in Experiment 1, with cue-type (go-consistent versus go-inconsistent on go trials; withhold-consistent versus withhold-inconsistent on withhold trials) and part (1–3) as within-subjects factors. In the go RT analysis, incorrect and missed go trials were removed.

	*Df1*	*Df2*	*Sum of squares effect*	*Sum of squares error*	*F*	*p*	*gen. η^2^*

**Go trials: go RT**							
Part	2	60	562108.20	342233.78	49.27	**<0.001**	0.342
Cue-type	1	30	476.52	5086.46	2.81	0.104	<0.001
Part by cue-type	2	60	513.92	6326.63	2.44	0.099	<0.001
**Go trials: *p*(correct)**							
Part	2	60	0.14	0.29	13.91	**<0.001**	0.149
Cue-type	1	30	0.00	0.05	0.61	0.440	0.001
Part by cue-type	2	60	0.00	0.03	0.12	0.887	<0.001
**Go trials: *p*(miss)**							
Part	2	60	0.17	0.18	29.77	**<0.001**	0.245
Cue-type	1	30	0.00	0.01	3.31	0.079	0.002
Part by cue-type	2	60	0.00	0.02	4.04	**0.026**	0.006
**Withhold trials: *p*(respond|signal)**							
Part	2	60	0.07	0.51	4.34	**0.027**	0.034
Cue-type	1	30	0.00	0.01	2.28	0.142	<0.001
Part by cue-type	2	60	0.00	0.03	1.11	0.336	<0.001

*Note*: *p*s < 0.05 are highlighted in bold.

The probability of correct go responses increased as a function of part (Table 2), but there was similarly no reliable main effect of cue-type and no reliable two-way interaction between part and cue-type. Bayesian analyses provided moderate support for the null hypothesis of no difference between the go-consistent and go-inconsistent cues (*BF_10_* = 0.25).

**Figure 3:**
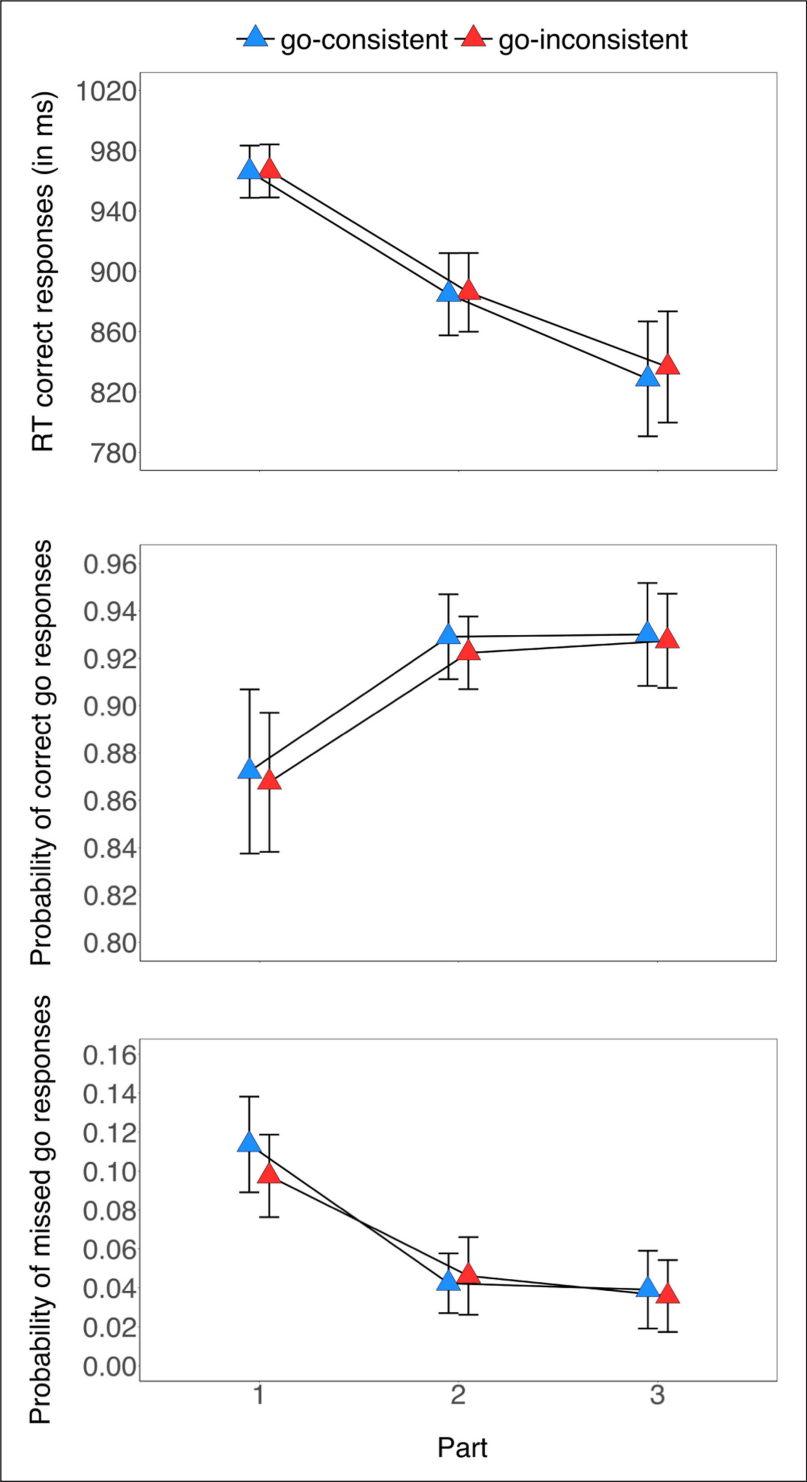
Reaction times for the correct go trials (upper panel), the probability of correct go responses (middle panel), and the probability of missed go responses (lower panel) in Experiment 1 as a function of cue-type (go-consistent, go-inconsistent) and part (1–3). Error bars reflect 95% confidence intervals.

The probability of missed go responses decreased as a function of part (Table 2), but the main effect of cue-type was not significant. The two-way interaction between part and cue-type was reliable: follow-up comparisons showed that the main effect of cue-type was reliable in part 1 (*p* = 0.019, g_av_ = 0.26, *BF_10_* = 2.57), but was not reliable in part 2 or 3 (*p*s ≥ 0.313, *BFs_10_* ≤ 0.31). As can be seen in Figure 3, the difference between the cue-types was very small (*M*_diff_ = 0.02) and was in the opposite direction to that predicted if participants acquired the the cue-signal location associations (i.e. numerically higher for the go-consistent cues than for the go-inconsistent cues). Thus, the *p*(miss) data directly argues against the hypothesis that participants learned to associate the go-consistent cues with the trained signal location.

Combined, there was consistent evidence across all three measures that go performance improved with training but no evidence that participants acquired the stimulus-signal associations.

#### Withhold analyses

**Figure 4:**
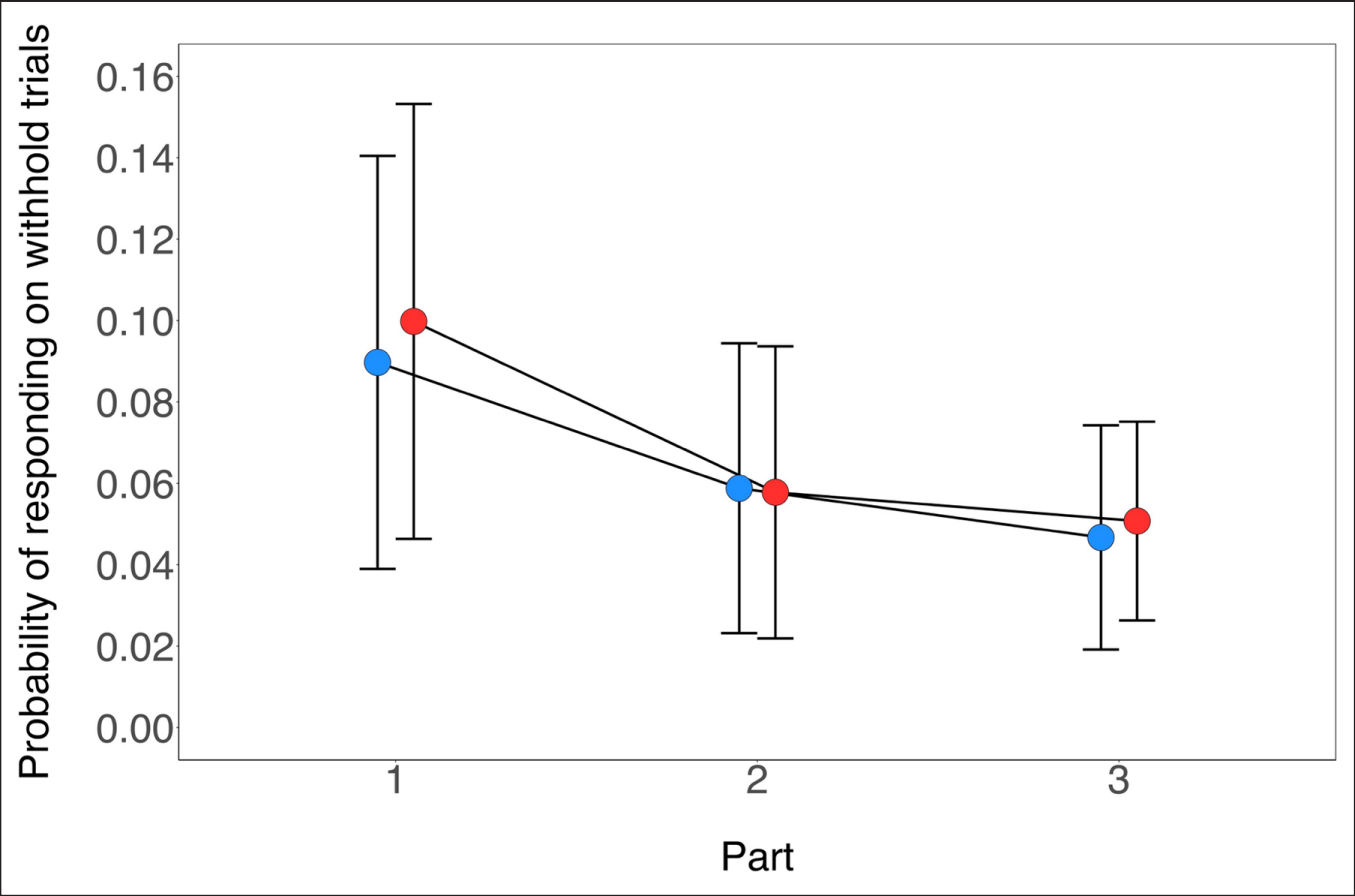
The probability of responding on withhold trials in Experiment 1 as a function of signal location-type (withhold-consistent, withhold-inconsistent) and part (1–3). Error bars reflect 95% confidence intervals. The probability of responding on withhold trials in Experiment 1 as a function of signal location-type (withhold-consistent, withhold-inconsistent) and part (1–3). Error bars reflect 95% confidence intervals.

As can be seen in Figure 4, the probability of responding on withhold trials decreased as a function of part (Table 2). However, there was no reliable difference between the cue-types and no reliable two-way interaction between part and cue-type (Table 2). The Bayes factor for the overall difference between the cue-types was 0.53 (i.e. anecdotal support for the null hypothesis).

#### Expectancy ratings analyses

**Figure 5:**
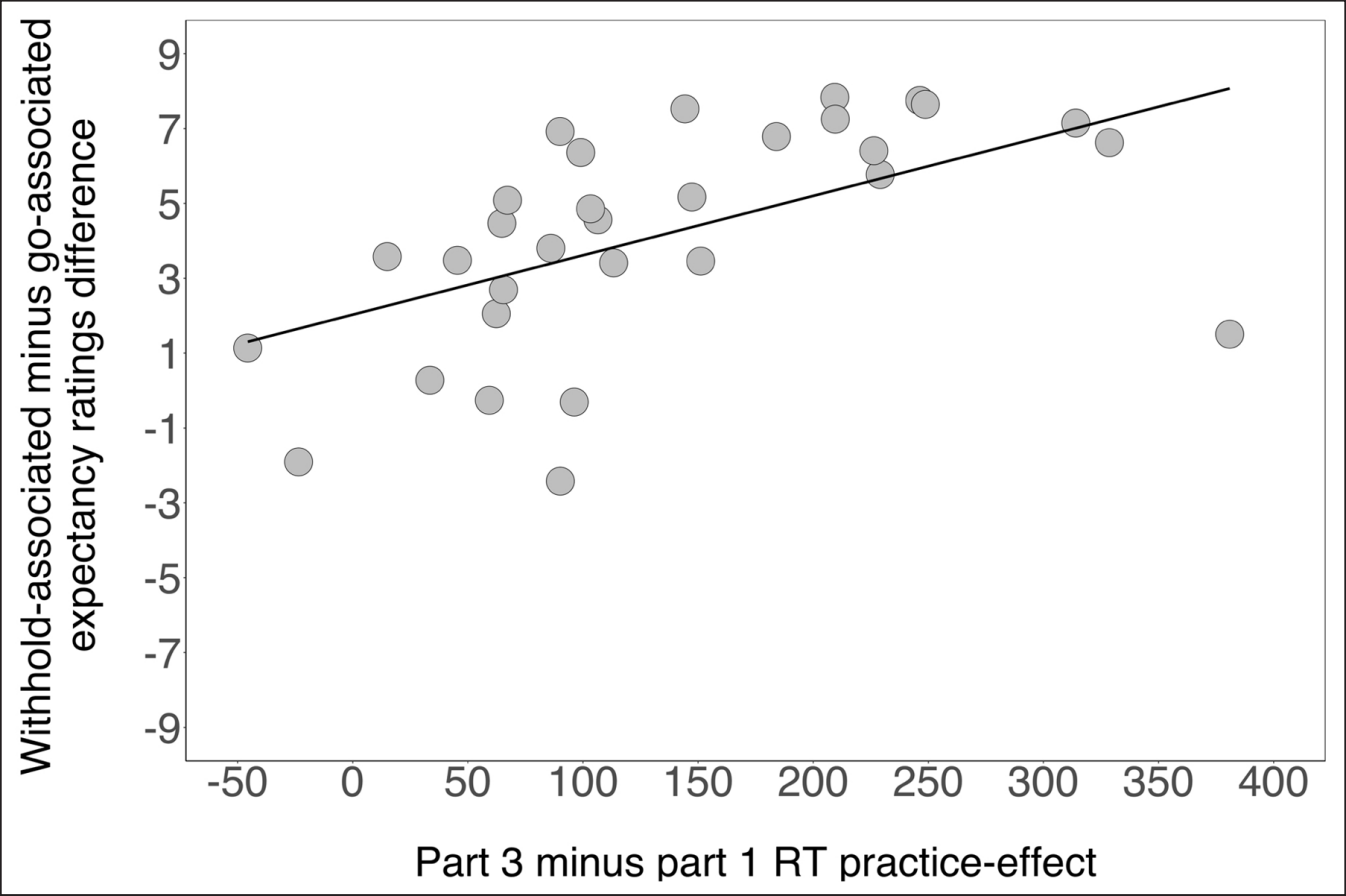
Correlation between the withhold- minus go-associated expectancy ratings difference and the part 3 minus part 1 RT practice-effect.

Following task completion, participants expected to withhold their response more for the withhold-associated cues (6.73) than for the go-associated cues (2.58), *F*(1, 30) = 60.79, *p* < 0.001, *gen. η^2^* = 0.628. The Bayes factor was 1225723, providing extreme evidence for the alternative hypothesis. Thus, participants were able to distinguish between the words on the basis of their association with the go and/or withhold goal. Furthermore, the expectancy ratings difference reliably correlated with the RT practice-effect on go trials (i.e. RT part 1 minus RT part 3), *r*(29) = 0.548, *p* = 0.001, *BF*_regression_ = 23.16: participants who expected to withhold their response less for the go-associated cues (and more for the withhold-associated cues) became faster throughout training (see Figure 5). This suggests that, alongside general practice-effects, the observed improvement in go performance with task practice reflects the acquisition of associations between the go-associated cues and responding (i.e. going). There was no reliable correlation between the stop-minus-go expectancy difference and the difference between part 1-minus-part 3 in the probability of responding on withhold trials, *r*(29) = –0.277, *p* = 0.132, *BF*_regression_ = 0.84. Thus, whilst the Bayesian regression provided strong evidence for a relationship between the expectancy ratings and the RT practice-effect, there was no support for the relationship between the expectancy ratings and task practice on withhold trials.

### Discussion

Go and withhold performance improved with task practice and participants generated expectancies that were consistent with the stimulus-withhold/go contingencies in play. These expectancies reliably correlated with the magnitude of the RT training-effect on go trials. However, there was no reliable evidence that participants acquired the associations between the cues and the spatial signal locations, or that consistent ‘contexts’ improved performance. The magnitude of the performance improvements was similar for the cues associated with the same signal location and for the cues associated with two signal locations with equal probability. The Bayesian analyses also provided support for the null hypothesis of no difference between these cue-types.

There are, however, some limits on the conclusions that can be drawn from this experiment. First, the withhold-associated cues were associated with the stop goal (or go goal for the go-associated cues) on 100% of presentations. Thus, it was not possible to compare the withhold-associated and go-associated cues during training. Whilst improvements with task practice most likely reflect some contribution of cue-specific withhold and/or go learning (see Verbruggen & Logan, 2008, for a discussion), they could also reflect general task learning. Second, although the use of coloured targets is common in the visual-attention literature (e.g. Woodman, 2013), a stimulus is more likely to capture attention if it is highly perceptually salient (e.g. if it is brightly coloured; Folk, Remington, & Johnston, 1992). Thus, it is possible that the use of coloured signals made it too easy to distinguish between the go and withhold signals on the basis of colour feature detection, diminishing the effect of prior learning on attention to the spatial locations of the signals. Third, whilst the high accuracy rates on go and withhold trials indicate that participants attended to the lateralised signals (most likely via a combination of shifts in spatial attention and colour feature detection) it is possible that the distance between the cue and the lateralised signals did not prompt large enough shifts in spatial attention to meaningfully mediate go or withhold learning. Fourth, as participants responded to the words on each trial via left or right keypresses, the spatial correspondence between the response to the words and the location of the withhold and go signals could have interfered with the acquisition of the signal-location contingencies (cf. the ‘Simon effect’; Simon & Rudell, 1967). Post-hoc analyses revealed support for this idea: the probability of correct responses on go trials was lower on incompatible trials (where the signal location of the go signal and the response hand were in opposite spatial locations; 0.89) than on compatible trials (where the signal location of the go signal and the response hand where in the same spatial location; 0.93), *t*(30) = 5.82, *p* < 0.001, g_av_ = 0.84. Similarly, there was also a reliable difference in the go RTs between the incompatible trials (897 ms) and the compatible trials (884 ms), *t*(30) = –5.85, *p* < 0.001, g_av_ = –0.20. Finally, it is possible that the interval between the presentation of the word and the onset of the signal (500 ms) did not provide participants with enough time to reorient their attention prior to the presentation of the withhold/go signal. We address these issues in Experiment 2.

## Experiment 2

In Experiment 2, we made several changes to the task design. First, the cues were not paired with withhold (or go) and the same signal location on all presentations (i.e. instead of a 100%-contingency mapping, we used a 80%-contingency mapping) to better index the effects of stimulus-withhold/go learning and stimulus-signal location learning on task performance. Second, the withhold and go signals differed in stimulus orientation (square, diamond) rather than in colour, to reduce the influence of stimulus saliency on signal detection (see Awh, Belopolsky, & Theeuwes, 2012) and make the signals more comparable with the ‘M’ and ‘W’ signals used by Elchlepp and Verbruggen (2017) in which measurable shifts in spatial attention occurred. Third, we increased the distance between the words and the signals. We expected that these modifications would make the go and withhold signals harder to distinguish, increasing the detection demands and encouraging more pronounced shifts in spatial attention. Fourth, to address the spatial correspondence ‘Simon effect’ issue, participants did not have to respond to the words, but were instead instructed to attend to them in anticipation for a recall test later in the experiment. Note that previous research has indicated that the presence of a choice task increases go response latencies but does not change the fundamental nature of the stopping process. For example, Logan, Cowan, and Davis (1984) showed that the same independent race model could account for stop-signal performance in both choice and simple RT tasks (even though the latency of the stop process was slightly different in both versions). Thus, the inhibitory control component of the tasks used in Experiment 1 and Experiment 2 was assumed to be largely the same. Fifth, we extended the duration between the onset of the cue and the onset of the withhold/go signal to 1000ms (cf. 500 ms in Experiment 1) to ensure that there was ample time for participants to reorient their attention (see e.g. Elchlepp, Best, Lavric, & Monsell, 2017).

We also collected more data to further inform our conclusions. In addition to the withhold expectancy ratings obtained in Experiment 1, participants also rated where they expected the withhold signal to appear on the screen (i.e. to the left or to the right of the cue). We also obtained eye-movement data in case the behavioural measures used in Experiment 1 were not sensitive enough to detect the effects of signal-location learning. However, consistent with similar studies (Best, McLaren, & Verbruggen, 2018; Verbruggen, Stevens, & Chambers, 2014), the eye-movement data did not inform our conclusions beyond the behavioural data. Therefore, we present the eye-movement data in the Supplementary Material only.

### Method

#### Participants

Thirty-two volunteers from the University of Exeter participated for monetary compensation (£10) or partial course credit (*M* = 20.25 years, *SD* = 3.13, 24 females, 31 right-handed). Two participants were replaced due to technical difficulties with the eye-tracking software; no participants were replaced for performance-related reasons. The target sample size and exclusion criteria were decided in advance of data collection to ensure that we had enough power (0.80) to detect medium-sized effects (Cohen’s *d* = 0.5) in the (one-tailed) within-subject comparisons.

#### Apparatus, stimuli, and procedure

The stimuli were presented on a 17-inch CRT monitor (screen size: 1024 × 768 pixels) using Psychtoolbox (Brainard, 1997). Eye movements were recorded throughout the experiment. An EyeLink 1000 Desktop Mount camera system (SR Research, Ottawa, Canada), calibrated before each block, tracked the gaze position of the right eye during the whole block (for further details, see Supplementary Material).

**Figure 6:**
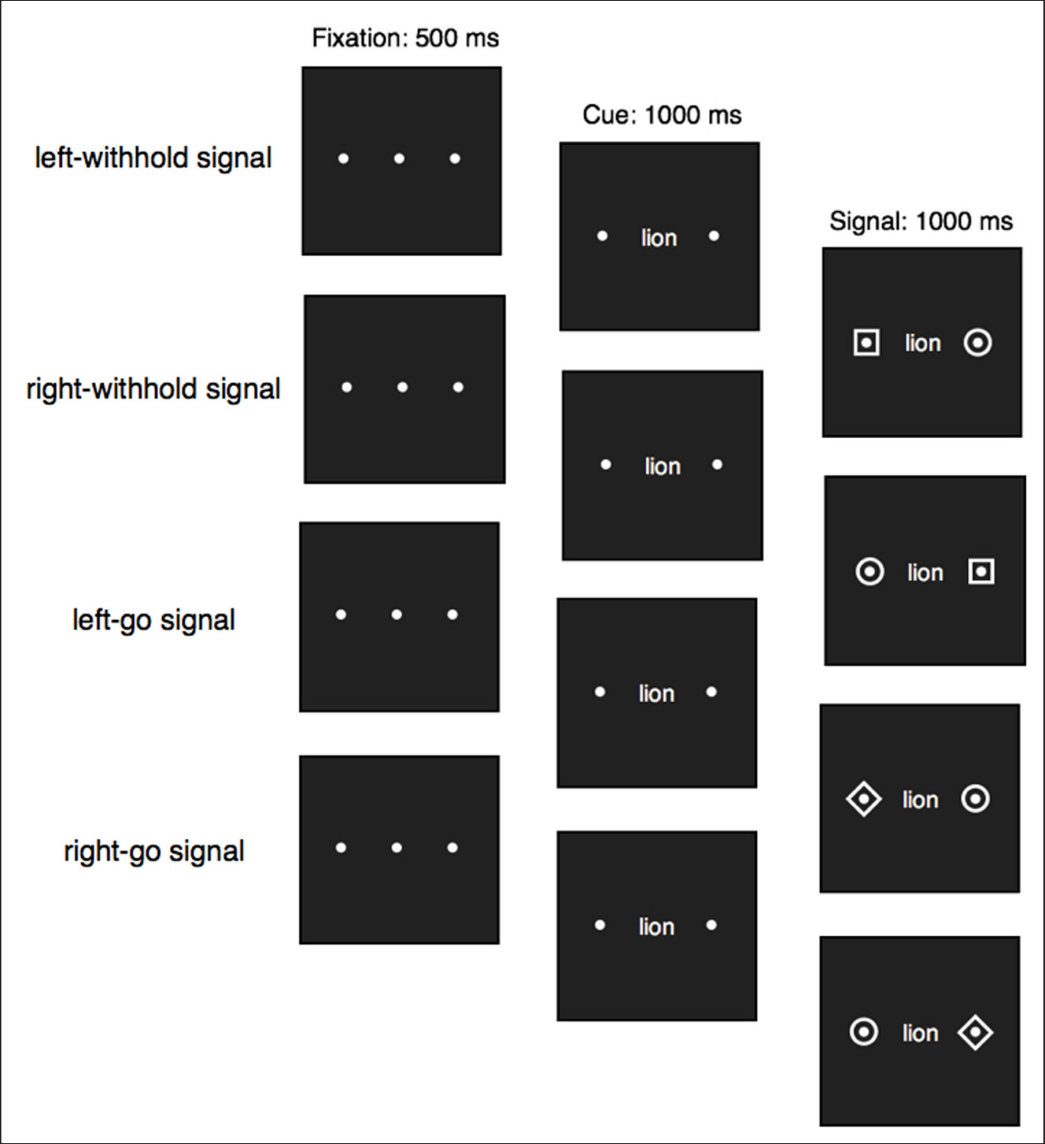
The left-withhold signal, right-withhold signal, left-go signal, and right-go signal trial structures in Experiment 2.

The cues consisted of a word in white lowercase font (Courier 16 point) presented on a black background (Figure 6). For half of the participants, the go signal was a diamond and the withhold signal was a square; for the other half, the go signal was a square and the withhold signal was a diamond (size: 24 × 24 pixels). Aside from the orientation, the go signal and withhold signal were identical. On half of all trials, the go signal or withhold signal was presented to the left of the word; on the other half, the go signal or withhold signal was presented to the right of the word (word-signal distance: 256 pixels or 6.8 cm). The distractor was always a circle, presented on the opposite side to the go/withhold signal (Figure 6). We randomly created three lists of 12 four-letter words (see Supplementary Material) for each participant. Two word lists were used in the main task, and the remaining word list was used in a recognition-ratings task following completion of the main task. To encourage participants to attend to the words, the presentation of the two word lists alternated on a block-by-block basis in the main task (e.g. wordlist 1, wordlist 2, wordlist 1, …). The experiment comprised three parts with four blocks per part. There were 120 trials per block. Each word was presented 10x per block; there were 7 presentations that were paired with a consistent response goal (e.g. withhold) and consistent signal location (e.g. left-withhold signal); 1 presentation that was consistent with the trained response (e.g. withhold) but inconsistent with the trained signal location (e.g. right-withhold signal); 1 presentation that was inconsistent with the trained response (e.g. go) but consistent with the trained signal location (e.g. left-go signal); and 1 presentation that was inconsistent with the trained response (e.g. go) and inconsistent with the trained signal location (e.g. right-go signal). As can be seen in Table 3, this resulted in four cue-types with equal proportions.

**Table 3:** The proportion of left withhold signal, right withhold signal, left go signal, and right go signal trials per block as a function of cue-type in Experiment 2. Note that there were 24 words used in the experimental task in total, with two lists of 12 words which alternated on a block-by-block basis (i.e. there were 12 words per block).

Cue-type	No. of words	% left withhold signal	% right withhold signal	% left go signal	% right go signal

**Withhold-left**	3	70	10	10	10
**Withhold-right**	3	10	70	10	10
**Go-left**	3	10	10	70	10
**Go right**	3	10	10	10	70

*Note*: The overall *p*(withhold-signal) was 0.50.

On each trial, three white fixation dots were presented marking the central, left, and right stimulus locations (Figure 6). After 500 ms, the central fixation dot was replaced with a word. After 1000 ms, one shape appeared around the fixation dot to the left of the word and one shape appeared around the fixation dot to the right of the word. The word and the signals remained on the screen for 1000 ms (hence, MAXRT was 2000 ms).

On go trials, participants responded by pressing the spacebar on a keyboard with their right index finger. Participants were instructed that “If one of the shapes is a [diamond/square] you should press the spacebar with your right index finger. If one of the shapes is a [square/diamond] you should withhold your response. You do not have long to respond when the shapes appear so you must respond as quickly as possible on go trials.” (for full instructions, see Supplementary Materials). Participants were informed that the aim of the study was to investigate memory for simple four-letter words and so they were instructed to attend to the words and try to remember as many as possible for a recall test following task completion. At the end of each block, participants received feedback on their mean RT on go trials, the number of incorrect key presses on go trials, the number of missed go responses, and the percentage of failed stops during that block. After 15 seconds, participants had to press any key to start the next block.

Following task completion, participants completed two ratings tasks. First, participants rated “How much do you expect to withhold your response when this word is presented?” (1 = *I definitely do not think this word indicates that I have to withhold my response* to 9 = *I definitely think this word indicates that I have to withhold my response*). Second, participants rated “Where do you expect the stop signal to appear on the screen?” (1 = *I definitely expect the stop signal to appear on the left* to 9 = *I definitely expect the stop signal to appear on the right*). The order of the words in both ratings tasks was randomised anew for each participant.

Finally, we probed memory for the words presented in the task. Note that these measures were obtained following the ratings tasks meaning that the recall of the words could be greater than if we obtained these measures immediately following task completion. First, participants performed a free recall test whereby they were instructed to write down as many words as they could remember from the main task in 80 seconds (for a similar procedure, see Snyder, Ashitaka, Shimada, Ulrich, & Logan, 2014). Second, participants were presented with the 24 words used in the main task and 12 novel words. Participants rated “How do you think that this word was presented in the main task?” (1 = *I definitely think this word was presented in the main task* to 9 = *I definitely do not think this word was presented in the main task*). Memory performance was not of primary interest in this paper, but we nevertheless report these data because they provide an indirect measure of the extent to which participants complied with the task instructions and attended to the words during the main task and/or expectancy ratings. Both measures confirmed that participants attended to the words: Participants recalled a mean average of 15 words (*SD* = 5.18) out of 24 words used in the task, and were able to distinguish between old words used in the task (1.17) and the novel words (8.36), *t*(31) = –30.44, *p* < 0.001, g_av_ = –9.02.

#### Analyses

**Figure 7:**
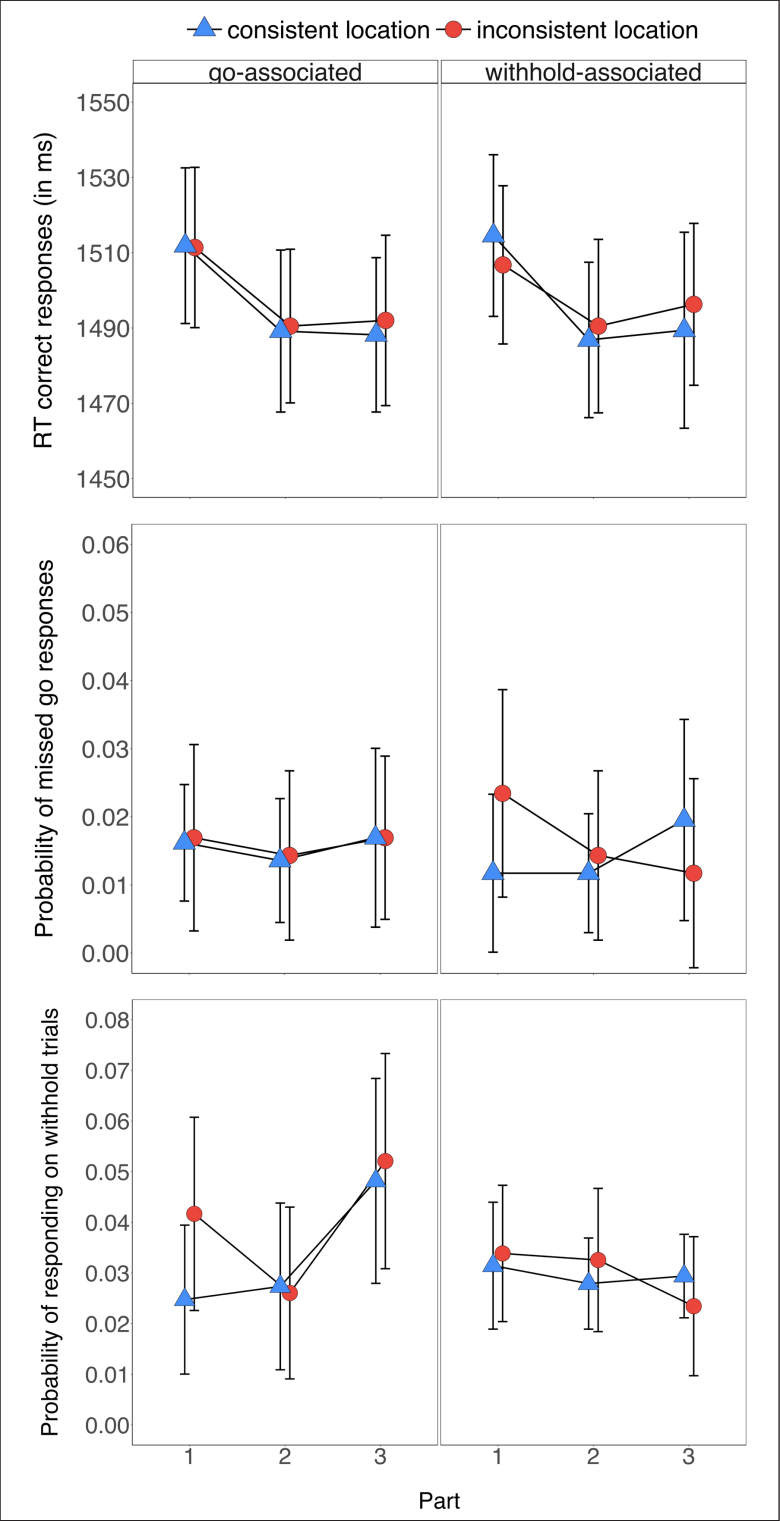
Reaction times for the correct go trials (upper panel), the probability of missed go responses (middle panel), and the probability of responding on withhold trials (lower panel) in Experiment 2 for the go-associated items (left panels) and for the withhold-associated items (right panels) as a function of signal location-type (consistent signal location, inconsistent signal location) and part (1–3). Error bars reflect 95% confidence intervals.

The behavioural analyses were identical to Experiment 1, except that the behavioural data was analysed as a function of withhold/go-type (withhold-associated cues, go-associated cues), signal-location (consistent with training, inconsistent with training), and part (1–3). We did not conduct inferential analyses on the *p*(miss) as values were very low. Figure 7 presents an overview of the descriptive statistics for the go RTs, *p*(miss), and the *p*(respond|signal); Table 4 presents an overview of the inferential statistics. Unlike in Experiment 1, we could not examine the *p*(correct) as participants responded with only one keypress on go trials (i.e. there was no choice task). The descriptive statistics for go and stop performance data can be found in Figure 7 and the inferential statistics can be found in Table 4.

**Table 4:** Overview of repeated measures Analyses of Variance in Experiment 2, with withhold/go-type (withhold-associated, go-associated), signal-location (consistent signal location, inconsistent signal location), and part (1–3) as within-subjects factors. In the go RT analysis, incorrect and missed go trials were removed.

	*Df1*	*Df2*	*Sum of squares effect*	*Sum of squares error*	*F*	*p*	*gen. η^2^*

**Combined analysis**							
Go trials: go RT							
Part	2	62	37140.47	73577.01	15.65	**<0.001**	0.027
Withhold/go-type	1	31	3.83	10330.14	0.01	0.915	<0.001
Signal-location	1	31	148.01	5362.99	0.86	0.362	<0.001
Part by withhold/go-type	2	62	311.89	16074.06	0.60	0.551	<0.001
Part by signal-location	2	62	1515.16	19538.16	2.40	0.099	0.001
Withhold/go-type by signal-location	1	31	9.63	10247.00	0.03	0.866	<0.001
Part by withhold/go-type by signal-location	2	62	535.26	26840.59	0.62	0.536	<0.001
Withhold trials: *p*(respond|signal)							
Part	2	62	0.01	0.08	2.26	0.112	0.009
Withhold/go-type	1	31	<0.001	0.04	3.47	0.072	0.007
Signal-location	1	31	<0.001	0.03	1.29	0.264	0.002
Part by withhold/go-type	2	62	0.01	0.08	5.54	**0.006**	0.020
Part by signal-location	2	62	0.00	0.06	0.98	0.368	0.003
Withhold/go-type by signal-location	1	31	0.00	0.05	0.58	0.451	0.001
Part by withhold/go-type by signal-location	2	62	0.00	0.06	0.92	0.402	0.003
**Withhold-associated cues only**							
Go trials: go RTs							
Part	2	62	17459.93	57184.14	9.47	**<0.001**	0.024
Signal-location	1	31	41.07	10713.65	0.12	0.733	<0.001
Part by signal-location	2	62	1900.69	37094.95	1.59	0.213	0.003
Withhold trials: *p*(respond|signal)							
Part	2	62	0.00	0.04	0.97	0.386	0.006
Signal-location	1	31	0.00	0.02	0.01	0.916	<0.001
Part by signal-location	2	62	0.00	0.05	0.63	0.534	0.005
**Go-associated cues only**							
Go trials: go RTs							
Part	2	62	19992.42	32466.92	19.09	**<0.001**	0.030
Signal-location	1	31	116.57	4896.34	0.74	0.397	<0.001
Part by signal-location	2	62	149.73	9283.80	0.50	0.609	<0.001
Withhold trials: *p*(respond|signal)							
Part	2	62	0.02	0.12	4.79	**0.012**	0.038
Signal-location	1	31	0.00	0.06	1.10	0.302	0.004
Part by signal-location	2	62	0.00	0.08	1.15	0.320	0.006

*p*s < 0.05 are highlighted in bold.

### Results

#### Go analyses

Responding became faster with task practice, as reflected by a reliable main effect of part (for inferential statistics, see Table 4). However, there was no reliable difference between the withhold-associated and go-associated cues and no reliable difference between trials in which the signals appeared in trained and untrained locations. The two-way interaction between withhold/go-type and the signal-location was also not reliable. Bayesian analyses provided moderate support for the null hypotheses of no difference between the withhold-associated cues and the go-associated cues, *BF_10_* = 0.19, and no difference between the consistent and inconsistent signal location trials, *BF_10_* = 0.28. Thus, performance benefited from non-specific task practice, but the stimulus-withhold/go contingencies and the stimulus-signal contingencies did not influence go task performance. The absence of signal learning on go trials is consistent with the exploratory analyses of the eye movement data: there was no evidence that participants fixated more in the the go-signal location that was consistent with training than in the go-signal location that was inconsistent with training, nor that they produced a greater number of fixations on trials where the go signal appeared in the inconsistent versus consistent location.

#### Withhold analyses

Consistent with the original memory-retrieval hypothesis, the probability of responding on withhold trials became lower for the withhold-associated cues than for the go-associated cues (Figure 7). This was reflected in the reliable two-way interaction between part and withhold/go-type (Table 4). Despite finding evidence that the stimulus-withhold/go associations influenced withhold performance, there was no reliable evidence that the signal location associations influence performance on withhold trials. The two-way interaction between withhold/go-type and signal-location was also not reliable. The corresponding Bayes factor for the difference between the consistent and inconsistent signal location trials provided support for the null hypothesis of no difference (*BF_10_* = 0.34). Thus, contrary to our earlier proposal, there was no evidence that performance on withhold trials was influenced by the stimulus-signal location associations. The absence of signal learning is on the probability of responding is consistent with the pattern of eye movements following withhold-signal presentation (Supplementary Material).

#### Expectancy ratings analyses

Following task completion, participants expected to stop more for the withhold-associated cues (5.13) than for the go-associated cues (4.56), *t*(31) = –2.45, *p* = 0.020, g_av_ = 0.65 (*BF_10_* = 2.45). The stop-minus-go expectancy ratings difference did not reliably correlate with the corresponding RT difference, *r*(30) = –0.081, *p* = 0.661, *BF*_regression_ = 0.36, nor the corresponding *p*(respond|signal) difference, *r*(30) = 0.16, *p* = 0.374, *BF*_regression_ = 0.46.

Analyses of the signal-location expectancy ratings showed no reliable main effect of location-type (left-associated cues vs. right-associated cues), *F*(1, 31) = 0.80, *p* = 0.377, *gen. η^2^* = 0.007. There was also no reliable location-rating difference between the withhold-associated cues (4.67) and the go-associated cues (4.94), *F*(1, 31) = 3.28, *p* = 0.080, *gen. η^2^* = 0.027, and no reliable two-way interaction between signal location-type and withhold/go-type (withhold-associated, go-associated), *F*(1, 31) = 3.66, *p* = 0.065, *gen. η^2^* = 0.026. Thus, the signal-location expectancies provide further evidence that the acquisition of the stimulus-go and stimulus-withhold associations was not reliably mediated via the signal location.

## General Discussion

In the present study, we investigated how learning influences signal-detection processes in a spatial response-inhibition task. Across two experiments, we found evidence that participants successfully learned to associate specific stimuli with the acts of either ‘withholding’ or ‘going’. These findings replicate and extend evidence of stimulus-stop learning in a novel response-inhibition task (see e.g. Best et al., 2016; Bowditch et al., 2016; Verbruggen & Logan, 2008b). Importantly, however, we found no reliable evidence that participants learned to associate the withhold-associated or go-associated cues with features of the withhold or go signals, including spatial location (despite electrophysiology work suggesting that signal detection played a crucial role in a similar spatial response-inhibition task; Elchlepp & Verbruggen, 2017).

In Experiment 1, there were no overall differences in withhold or go task performance and no reliable difference in the magnitude of the practice-effect between the cues that were associated with withhold or go signals in the same location (e.g. always to the left of the cue or always to the right of the cue) and the cues that were associated with withhold or go signals in two locations (e.g. to the left and to the right of the cue with equal probability). Bayesian analyses provided support for the null hypothesis of no difference between these cue-types.

In Experiment 2, a reliable difference emerged between the withhold-associated cues and the go-associated cues in the probability of responding on withhold trials, suggesting that participants learned the withhold/go associations. However, consistent with Experiment 1, we found no reliable difference between task performance on trials in which the signal location was consistent with training and trials in which the signal location was inconsistent with training. Bayesian analyses also provided support for the null hypothesis of no difference between these trials in go RTs and in the signal-location expectancy ratings following task completion. Therefore, despite evidence of go/withhold learning in both experiments, we conclude that participants did not learn to associate the cues with the spatial signal location of the withhold or go signal in this paradigm.

We previously proposed that there are at least two pathways through which learning in response-inhibition tasks could influence behaviour (Verbruggen, Best, et al., 2014; Figure 1). Specifically, when the stop or withhold signal remains the same throughout training, we suggested that participants could acquire associations between a stimulus and a representation of whatever stop/withhold signal (or no-go category) was used in that task. Based on a review of various measures of learning in stop-signal paradigms, we suggested that this indirect signal-mediated pathway would take precedence in traditional response-inhibition paradigms (Verbruggen, Best, et al., 2014). This idea is consistent with other studies highlighting the importance of signal-detection processes in response-inhibition tasks (e.g. Elchlepp & Verbruggen, 2017).

Based on the absence of stimulus-signal learning in the present study, our hypothesis that response inhibition would *always* operate through the indirect pathway when the representation of the stop or withhold signal remains the same throughout training seems unlikely. It might be possible to salvage the indirect pathway if we assume that response inhibition is mediated only via non-spatial features of the signal or in tasks that demand larger shifts in spatial attention. There is some indirect evidence to support the suggestion that non-spatial features of the signal mediates the stimulus-stop contingencies. Previous research has indicated, for example, that the effects of stimulus-stop learning are stronger when stop-associated stimuli are paired with multiple coloured stop signals compared with when stop-associated stimuli are paired with a single coloured stop signal (Bowditch, Verbruggen, et al., 2016). These findings are consistent with the idea that participants learned ‘direct’ stimulus-stop associations when presented with several representations of the stop signal and ‘indirect’ signal-mediated associations when presented with a single representation of the stop signal throughout the task (see Figure 1). Similarly, research in the visual attention literature has shown that attentional selection can occur on the basis of independent features, with colour and shape gaining priority over spatial location (Andersen, Müller, & Hillyard, 2009; Kasten, & Navon, 2008; Saenz, Buracas, & Boynton, 2002).

Previous studies have shown that implicit learning of spatial information occurs only when participants attend to predictive information (Jiang & Chun, 2001; Jiang & Leung, 2005; see also Turk-Brown, Jungé, & Scholl, 2005). Participants had to attend to the go and withhold signals in the present study, but it is possible that the attentional demands were not high enough and that the displays were not complex enough for learning effects to emerge. Note, however, that it seems likely that at least some shifts of spatial attention were required for performance of the present task. As discussed above, the presentation of similar lateralised ‘withhold’ signals (the letters ‘M’ and ‘W’) in earlier work elicited measurable shifts in spatial attention to the periphery in order to facilitate signal detection, as shown in early perceptual event-related potential components (Elchlepp & Verbruggen, 2017). Furthermore, we further increased the distance between the central cue and the lateralised signals from Experiment 1 to Experiment 2, to encourage shifts of spatial attention during signal detection and used shapes rather than colours to decrease signal detection on the basis of non-spatial features. The eye movement data (presented in the Supplementary Materials) indicates that participants fixated on the signal and distractor locations on a small proportion of the average trial. Importantly, they did not discriminate much between the actual (trained) signal and the distractor on the opposite side (only at the beginning of the experiment, there were some numerical differences in the expected direction). Future research should examine whether stop learning is mediated via the spatial location of the signals in a response-inhibition task that demands larger, more pronounced shifts in spatial attention.

Importantly, even if it does turn out that the effects of stimulus-signal learning are stronger for some stimulus attributes than others, the present research nevertheless shows that participants do not always ‘exploit’ the available spatial signal associations during stimulus-stop learning (even in variants of a task in which spatial shifts of attention have been shown to contribute to intra-individual differences in stopping performance; Elchlepp & Verbruggen, 2017). This informs our understanding of the cognitive architecture of stop learning, suggesting that the indirect signal-mediated pathway operates in much more restricted conditions than we originally hypothesised.

The main prediction with respect to the indirect pathway was that the retrieval of stimulus-signal associations would prime the representation or features of the stop signal rather than the stop goal or stop response (see also Best et al., 2016; Bowditch, Verbruggen, et al., 2016; Verbruggen, Best, et al., 2014). We suggested that this would result in a lower probability of responding for withhold-associated cues than for go-associated cues but, unlike the retrieval of direct stimulus-stop associations, there would be little or no difference between the withhold-associated and go-associated cues in go reaction times. We previously interpreted this behavioural pattern of results (i.e. learning affecting *p*(respond|signal) but not go RT) as evidence for the indirect pathway (Verbruggen, Best, et al., 2014). However, we observed this pattern of findings in Experiment 2 but did not find any reliable differences in performance between the consistent and inconsistent signal location cues. This suggests that an effect of learning on the probability of responding but not on go RTs is not necessarily indicative of learning via the indirect pathway. We suggest the following alternative interpretation of this pattern of results.

**Figure 8:**
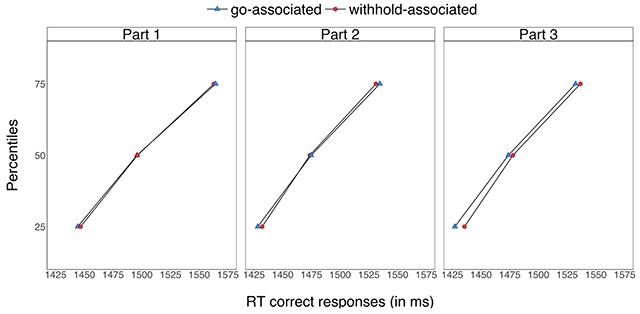
Go RTs (in ms) for the withhold- and go-associated cues as a function of percentile in Experiment 2. The two-way interaction between cue-type (withhold-associated, go-associated) and percentile (25th, 50th, 75th) was reliable, *F*(2, 62) = 3.52, *p* = 0.048, *gen. η^2^* < 0.001. Follow-up tests showed that the main effect of cue-type was reliable in the 25th percentile (*p* = 0.001, g_av_ = 0.107) but was not reliable in the 50th (*p* = 0.551, g_av_ = 0.018) and 75th (*p* = 0.916, g_av_ = –0.004) percentiles. Bayesian analyses also showed strong support for the alternative hypothesis of a cue-type effect in the 25th percentile (*BF_10_* = 21.21) and moderate support for the null hypothesis of no cue-type effect in the 50th (*BF_10_* = 0.22) and 75th (*BF_10_* = 0.19) percentiles.

Response slowing can be due to various factors, including strategic waiting (e.g. Bissett & Logan, 2011) or retrieval of previous stop episodes. Strategic adjustments and memory retrieval may have non-additive effects on performance. Specifically, general strategic adjustments and response slowing can fluctuate over trials. On trials in which subjects generally wait (regardless the identity of the word), we may observe no additional effects of retrieving the stimulus-stop associations as responding is already slowed. However, on trials in which there is less general slowing, item-specific effects may emerge. Importantly, this could explain why learning effects are observed in *p*(respond|signal) but not mean go RT. After all, mean RT is based on the entire go distribution (including the slower right tail), whereas the *p*(respond|signal) is primarily influenced by the fastest part of the RT distribution. For example, increasing the skew of an RT distribution will influence mean RT but it will not much influence *p*(respond|signal) (Verbruggen, Chambers, & Logan, 2013). We conducted an exploratory analysis to test this idea in the present study. As can be seen in Figure 8, responding was slower for the withhold-associated words than for go-associated words at the faster part of the RT distribution (25th percentile) but not at the middle and slower parts (50th and 75th percentiles). Furthermore, consistent with our predictions, this trend emerges across task performance. Inspired by this finding, we also conducted similar analyses on our previous stop-learning studies and found a consistent pattern of results (e.g. see Verbruggen, Best, et al., 2014, Experiment 2; Best et al., 2016, Experiment 4). Thus, evidence of stop learning in the probability of responding but not in go RTs may not reflect the acquisition qualitatively different kinds of learning after all, but may merely be an artifact of focusing only on the fastest trials that escape inhibition [i.e. *p*(respond|signal)] versus the whole RT distribution. As the rationale for this analysis was post-hoc in the present study, future research is required to test this idea directly.

## Conclusions

Taken together, the experiments presented in this paper replicate and extend evidence showing that participants can acquire item-specific associations between the acts of stopping and going. However, across two experiments, we find no evidence to support the idea that participants acquired the associations between specific stimuli and the spatial location of the withhold or go signal. Thus, contrary to our previously proposed general architecture of stop learning (Verbruggen, Best, et al., 2014), these findings suggest that learning in response-inhibition tasks is not *always* mediated via signal-detection processes when the spatial representation of the stop or withhold signal remains the same throughout training.

## Data Accessibility Statement

All data files and analysis scripts are deposited in the Open Science Framework (https://osf.io/j6tk9/?view_only=329b1312f7524ef58bb43050e5100cd0).

## Additional File

The additional file for this article can be found as follows:

10.5334/joc.73.s1 Supplementary Material. Words used in Experiments 1 & 2, on-screen task instructions and eye-movement data analyses.

## Footnotes

Note that Elchlepp and Verbruggen (2017) used the letters ‘M’ and ‘W’ as lateralised signals in their task. In the present study, we used coloured dots (Experiment 1) and shapes (Experiment 2) as lateralised signals.
