# Supplementary material for: Does Learning Influence the Detection of Signals in a Response-Inhibition Task?
Source: J Cogn. 2019 Jul 31;2(1):19. doi: 10.5334/joc.73 (PMC6676923; doi:10.5334/joc.73)
Supplement: Supplementary Material. — Words used in Experiments 1 & 2, on-screen task instructions and eye-movement data analyses. [file joc-2-1-73-s1.pdf]

SUPPLEMENTARY MATERIAL

Words used in Experiment 1

We used 112 words (56, five-letter, 56 six-letter) in Experiment 1. Half of the words referred to natural objects, the other half referred to human-made objects. The natural and human-made words were matched on word frequency (SUBTLEXWF, Brysbaert & New, 2009; *M*= 2.39, *SD*= 0.51).

Natural: sheep, eagle, otter, camel, hippo, grape, shark, snail, raven, chimp, horse, moose, llama, squid, chick, onion, lemon, panda, maple, snake, finch, sloth, apple, acorn, peach, cedar, basil, rhino, monkey, turtle, lizard, peanut, iguana, banana, papaya, orchid, turkey, tomato, donkey, walnut, potato, baboon, carrot, rabbit, falcon, salmon, weasel, pepper, ferret, shrimp, walrus, pigeon, celery, garlic, gibbon, radish.

Human-made: brick, scarf, canoe, cabin, diary, label, whisk, ferry, quill, leash, apron, spoon, shawl, bench, banjo, skirt, penny, torch, arrow, swing, medal, couch, piano, brush, wagon, towel, buggy, badge, brooch, crayon, cement, teacup, cradle, pajama, sandal, seesaw, jigsaw, napkin, paddle, helmet, funnel, statue, sleigh, eraser, jumper, stereo, anchor, cinema, poster, violin, candle, pillow, dinghy, bucket, guitar, corset.

Words used in Experiment 2

There were 36 words in Experiment 2. Words were randomly allocated to three word lists of 12 words each anew for each participant. Two of the word lists were used in the main task, the remaining word list was used in the recognition ratings following task completion (For

more details, see Procedure). Participants were not required to respond to the words in this experiment, so all words referred to natural objects.

Words: mule, calf, kiwi, wren, swan, newt, leaf, stag, lime, clam, crow, flea, pear, wasp, moth, rice, plum, germ, slug, dove, toad, boar, crab, pony, deer, worm, lamb, goat, frog, hawk, tree, lion, wolf, duck, bull, bear.

On-Screen Task Instructions

Experiment 1

The go, withhold, and distractor colours remained the same throughout Experiment 1, but were counterbalanced across participants (e.g. P1: blue = go, pink = withhold, yellow = distractor; P2: yellow = go, blue = withhold, pink = distractor). Note also that half of the participants had to press the 'c' key (with their left index finger) when the word referred to a natural object and the 'm' key (with their right index finger) when the word referred to a human-made object. This mapping was reversed for the other half of participants. Thus, the square brackets denote instructions that were varied for each participant depending on the assigned signal and response mappings.

"On every trial, you will see three black dots on the screen. After a delay, a word will replace the dot in the centre of the screen. The dots on the left and on the right of the word will remain on the screen. On most trials, one of the dots will turn [pink/blue/yellow]. On these trials, you have to decide whether the word refers to a natural or a human-made object. Press the ["c" /"m"] key when the word refers to a natural object and the ["m" /"c"] key when it refers to a human-made object. You should respond as quickly and as accurately as possible on these trials. However, on a selection of trials one of the dots will turn [blue/pink/yellow]. This indicates that you have to stop your response. You should not press any key on these trials."

Experiment 2

For half of the participants in Experiment 2, the go signal was a diamond and the withhold

signal was a square; for the other half, the go signal was a square and the withhold signal was a diamond. Thus, the square brackets denote instructions that were varied for each participant depending on the assigned signal mapping.

"In this experiment, we are interested in your memory for simple four-letter words during performance of a reaction time task. You will first complete a word reaction time task. On every trial, you will see a word in the centre of the screen. You must pay attention to this word. Your task is to remember as many of these words as you can. You will be tested on your recall of these words at the end of the task. After a short delay following the word presentation, one shape will appear on the left and one shape on the right of the word. If one of the shapes is a [diamond/square] you should press the spacebar with your right index finger. If one of the shapes is a [square/diamond] you should withhold your response. You do not have long to respond when the shapes appear so you must respond as quickly as possible on go trials."

Eye-Movement Data

The gaze position of the right eye was tracked throughout task performance to further measure shifts of attentional focus within each trial. Previous research has shown that gaze position can be used as a measure of attention in learning tasks (e.g. Rehder & Hoffman, 2005). The signal-learning account makes several predictions about the pattern of eye movements following signal presentation: (1) the number of fixations would become greater when the location of the presented signal was inconsistent with training (vs. consistent with training), reflecting the requirement to reorient spatial attention (away from the region where the signal usually occurs towards the region where it actually occurs); (2) the proportion of fixations on the actual signal location would become greater on consistent location trials than on inconsistent location trials; (3) the proportion of fixations on the opposite signal would become greater on inconsistent location trials than on consistent location trials, reflecting the allocation of spatial attention to the location of the trained signal.

An EyeLink 1000 Desktop Mount camera system (SR Research, Ottawa, Canada) was calibrated at the beginning of each block (there were 120 trials between calibrations). Note that the numbers of trials (and time durations) between calibrations using this system is consistent with previous research utilising eye-tracking in response-inhibition tasks (Verbruggen, Stevens, et al., 2014). The gaze position of the right eye was tracked throughout each block (sampling rate: 500 Hz). The EyeLink was calibrated and controlled via Psychtoolbox (Cornelissen, Peters, & Palmer, 2002).

The eye-movement data were exported using the Eyelink Data Viewer (SR Research, Ottawa, Canada). We integrated the file with information about all fixations and the file with trial sequence information using R (R Development Core Team, 2014). Fixations were

defined by the Eyelink software as any period that was not a blink or saccade. No minimum duration was set for fixations. Participants were excluded from the analyses when no fixation was registered at the beginning of the trial event (i.e. the presentation of the word) on more than 15% of trials as this could indicate suboptimal eye-movement registration. Based on this criterion, four participants were excluded from the eye-movement analyses. The inclusion of these participants did not substantially alter the overall pattern of behavioural results. All fixations that were off screen were excluded (0.2%).

We analysed eye movements in the interval following signal presentation. Eye-movements made 471 ms (mean RT on correct and incorrect go trials) after the presentation of the signal were excluded. We analysed the number of fixations and the proportions of fixations in four predefined regions of interest (see Figure S1): the word region, the actual signal location, the opposite (i.e. control) signal location, and an outside region (for all other fixations). Regions of interest were defined as 100 × 100 px squares around the central word region, the left signal region, and the right signal region. For each trial, the left and right signal locations were coded as the actual vs. opposite signal locations. Thus, there was always an actual signal region and an opposite signal region on each trial.

We conducted separate ANOVAs on the number of fixations, the proportion of fixations in the actual signal region, and the proportion of fixations in the opposite signal region with withhold/go-type, signal-location (consistent with training, inconsistent with training), signal-type (withhold signal, go signal), and part (1-3) as within-subjects factors.

For completeness, we also present the proportions of the fixations in the interval between word presentation and go/withhold-signal presentation that fell within four regions

(the word region, the trained signal location, and the opposite signal location, and the outside region) in Figure S6.

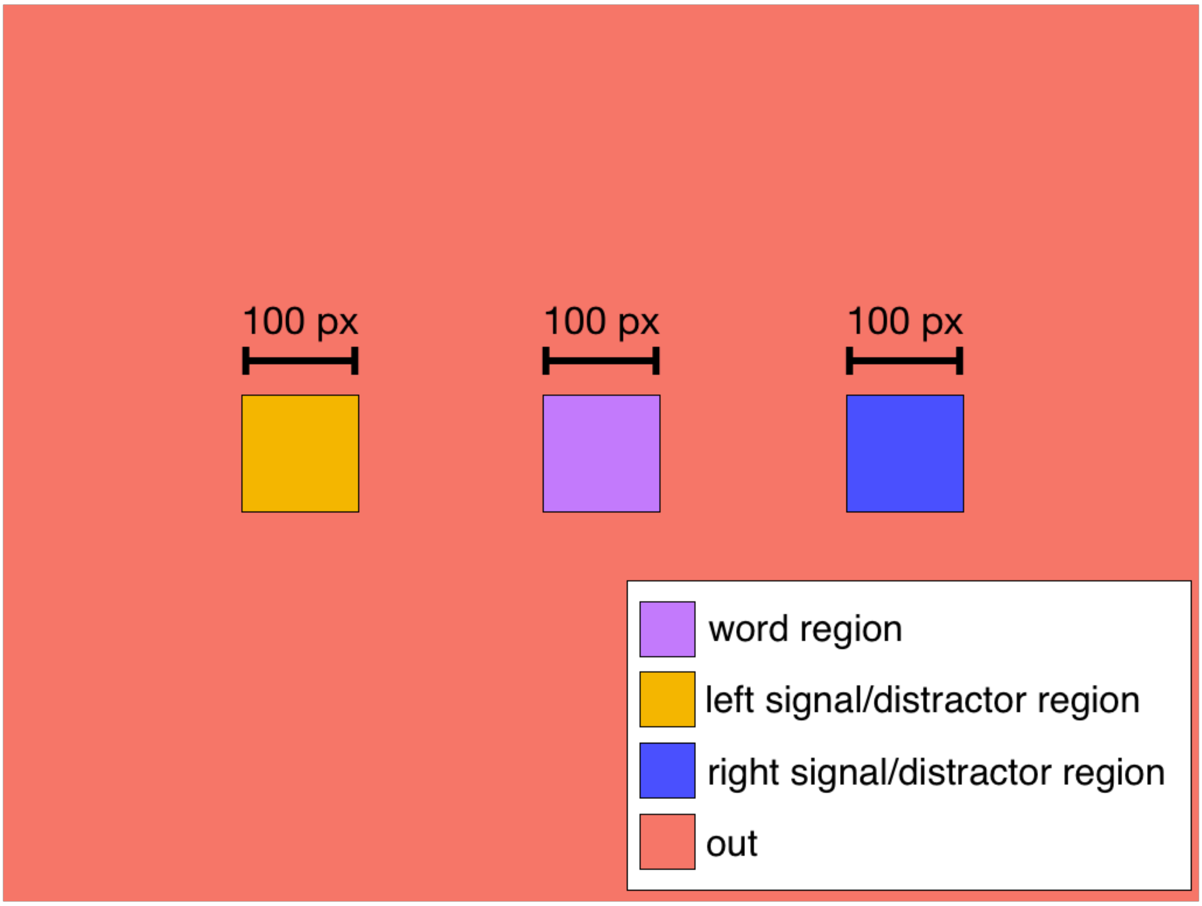

Figure S1: To analyse fixation location, we predefined 3 regions (squares): a central region around the word cue, a region around the left signal location, and a region around the right signal location. All fixations that did not occur within these regions were coded as 'out'. The size of each square is in pixels because pixel coordinates were used for registration of fixation location. Screen size: 1024 x 768 pixels.

The number of fixations in the signal interval decreased with task practice, as reflected by the reliable main effect of part (Tables S1-S2). However, there was no reliable difference between the trials in which the signal location was consistent with training and the trials in which the signal location was inconsistent with training (Table S2). Bayesian

analyses showed moderate support for the null hypotheses of no difference between the consistent signal location trials and the inconsistent signal location trials (BF_{10} = 0.26). Thus, there was no support for our first hypothesis that the number of fixations would become greater on inconsistent location trials compared with consistent signal location trials.

The proportion of fixations on the actual signal region decreased with task practice (a reliable main effect of part; Table S3). However, there was no reliable difference between the proportion of fixations on the actual signal region on consistent location trials versus inconsistent location trials, and no reliable evidence that a difference between these trial types emerged with task practice (i.e. no reliable two-way interaction between part and signal-location; Table S3). This overall pattern was the same following the presentation of the go- and withhold-signals and for the go-associated and withhold-associated cues (see Figures S2-S5). Bayesian analyses revealed anecdotal support for the null hypothesis of no difference between the consistent and inconsistent trials (BF_{10} = 0.50). Thus, there was no support for our second hypothesis that attention to the actual signal location would be influenced by stimulus-signal learning.

Analyses of the proportion of fixations in the opposite signal region similarly revealed no reliable difference between the inconsistent location trials and the consistent location trials. There was also no reliable two-way interaction between signal location and part (Table S3). Again, this pattern was the same following the presentation of the go- and withhold-signals and for the go- and withhold-associated words (see Figures S2 and S3). Bayesian analyses provided 'moderate' support for the null hypothesis of no difference between the consistent and inconsistent trials (BF_{10} = 0.27).

Combined, there was no evidence that learning influenced attention to the signals, as measured by eye movements on go or withhold trials. This is consistent with the behavioural data presented in the main text.

	Fixation			
	consistent signal location		*inconsistent signal location*	
	M	***SD***	***M***	***SD***
**Go signal**				
Part 1				
Go-associated	1.84	0.49	1.85	0.48
Withhold-associated	1.87	0.52	1.82	0.45
Part 2				
Go-associated	1.59	0.31	1.59	0.34
Withhold-associated	1.58	0.34	1.62	0.35
Part 3				
Go-associated	1.60	0.30	1.56	0.36
Withhold-associated	1.61	0.35	1.57	0.32
**Withhold signal**				
Part 1				
Go-associated	1.90	0.58	1.92	0.59
Withhold-associated	1.92	0.55	1.91	0.58
Part 2				
Go-associated	1.70	0.41	1.67	0.35
Withhold-associated	1.66	0.33	1.68	0.35
Part 3				
Go-associated	1.65	0.35	1.64	0.33
Withhold-associated	1.65	0.32	1.62	0.33

Table S1: Overview of the number of fixations in the interval after the stop signal for each withhold/go-type (withhold-associated, go-associated), signal-location (consistent signal location, inconsistent signal location), signal-type (withhold signal, go signal), and part (1-3). *M* = mean; *SD* = standard deviation.

	Df1	*Df2*	*Sum of squares effect*	*Sum of squares error*	*F*	*p*	*gen.* η^2
**Number of fixations**							
Withhold/go-type	1	27	0.00	0.37	0.00	0.968	< 0.001
Signal-type	1	27	0.71	0.78	24.40	**< 0.001**	0.006
Part	2	54	9.54	11.51	22.40	**< 0.001**	0.079
Signal-location	1	27	0.01	0.43	0.53	0.473	< 0.001
Withhold/go-type by signal-type	1	27	0.01	0.27	0.62	0.436	< 0.001
Withhold/go-type by part	2	54	0.00	0.79	0.04	0.956	< 0.001
Signal-type by part	2	54	0.02	1.25	0.47	0.627	< 0.001
Withhold/go-type by signal-location	1	27	0.00	0.44	0.02	0.897	< 0.001
Signal-type by signal-location	1	27	0.00	0.27	0.37	0.549	< 0.001
Part by signal-location	2	54	0.04	0.69	1.48	0.237	< 0.001
Withhold/go-type by signal-type by part	2	54	0.00	0.78	0.14	0.867	< 0.001
Withhold/go-type by signal-type by signal-location	1	27	0.00	0.41	0.03	0.863	< 0.001

Withhold/go-type by part by signal-location	2	54	0.07	0.86	2.17	0.128	0.001
Signal-type by part by signal-location	2	54	0.02	0.71	0.79	0.460	< 0.001
Withhold/go-type by signal by signal-location	2	54	0.00	0.78	0.17	0.848	< 0.001

Table S2: Overview of Analyses of Variance performed to compare number of fixations following signal presentation. Withhold/go-type (withhold-associated, go-associated), signal-location (consistent location, inconsistent location), signal-type (withhold signal, go signal), and part (1-3) were within-subjects factors. *p*s < 0.05 are highlighted in bold.

	Df1	*Df2*	*Sum of squares effect*	*Sum of squares error*	*F*	*p*	*gen.* η^2
**Proportion of fixations in the actual signal region**							
Withhold/go-type	1	27	0.01	0.05	4.93	**0.035**	0.001
Signal-type	1	27	0.03	0.07	11.90	**0.002**	0.003
Part	2	54	1.29	0.88	39.56	**< 0.001**	0.115
Signal-location	1	27	0.00	0.03	2.04	0.165	0.000
Withhold/go-type by signal-type	1	27	0.00	0.02	0.01	0.905	0.000
Withhold/go-type by part	2	54	0.01	0.09	2.49	0.098	0.001
Signal-type by part	2	54	0.09	0.10	24.54	**< 0.001**	0.009
Withhold/go-type by signal-location	1	27	0.00	0.05	0.01	0.934	0.000
Signal-type by signal-location	1	27	0.00	0.07	1.03	0.319	0.000
Part by signal-location	2	54	0.01	0.07	2.85	0.066	0.001
Withhold/go-type by signal-type by part	2	54	0.00	0.07	0.93	0.402	0.000
Withhold/go-type by signal-type by signal-location	1	27	0.00	0.05	0.33	0.572	0.000
Withhold/go-type by part by signal-location	2	54	0.00	0.07	0.12	0.885	0.000
Signal-type by part by signal-location	2	54	0.00	0.13	0.49	0.615	0.000
Withhold/go-type by signal by signal-location	2	54	0.01	0.09	2.68	0.078	0.001
**Proportion of fixations in the distractor region**							
Withhold/go-type	1	27	0.00	0.06	0.26	0.615	0.000
Signal-type	1	27	0.04	0.07	16.76	**< 0.001**	0.006
Part	2	54	0.00	0.45	0.15	0.768	0.000
Signal-location	1	27	0.00	0.02	0.67	0.419	0.000
Withhold/go-type by signal-type	1	27	0.00	0.03	2.18	0.152	0.000
Withhold/go-type by part	2	54	0.01	0.09	2.20	0.123	0.001
Signal-type by part	2	54	0.07	0.12	14.98	**< 0.001**	0.009
Withhold/go-type by signal-location	1	27	0.00	0.06	0.25	0.621	0.000
Signal-type by signal-location	1	27	0.00	0.05	2.15	0.154	0.001
Part by signal-location	2	54	0.00	0.08	0.91	0.403	0.000
Withhold/go-type by signal-type by part	2	54	0.02	0.07	7.97	**0.002**	0.003
Withhold/go-type by signal-type by signal-location	1	27	0.00	0.03	0.00	0.994	0.000
Withhold/go-type by part by signal-location	2	54	0.00	0.08	0.41	0.664	0.000
Signal-type by part by signal-location	2	54	0.00	0.08	0.26	0.769	0.000
Withhold/go-type by signal by signal-location	2	54	0.01	0.07	2.72	0.083	0.001

Table S3: Overview of Analyses of Variance performed to compare the proportions of fixations following signal presentation. Separate ANOVAs were conducted on the proportion of fixations in the actual signal region and on the proportion of fixations in the opposite signal region. Withhold/go-type (withhold-associated, go-associated), signal-location (consistent location, inconsistent location), signal-type (withhold signal, go signal), and part (1-3) were within-subjects factors. ps < 0.05 are highlighted in bold.

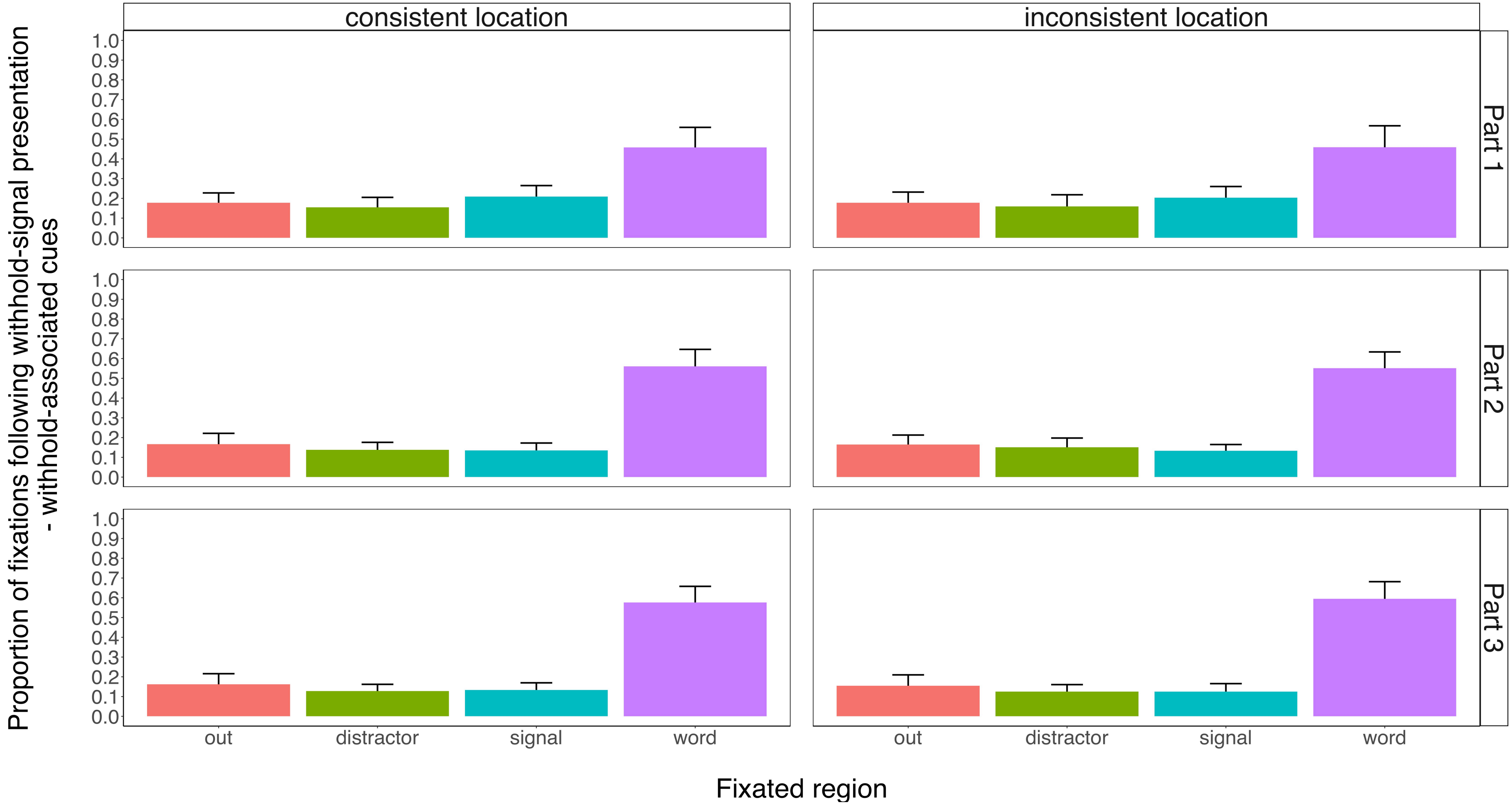

Figure S2: The proportion of fixations following withhold-signal presentation for withhold-associated cues as a function of region (word, signal, distractor, out), part (1-3), and signal location (consistent, inconsistent).

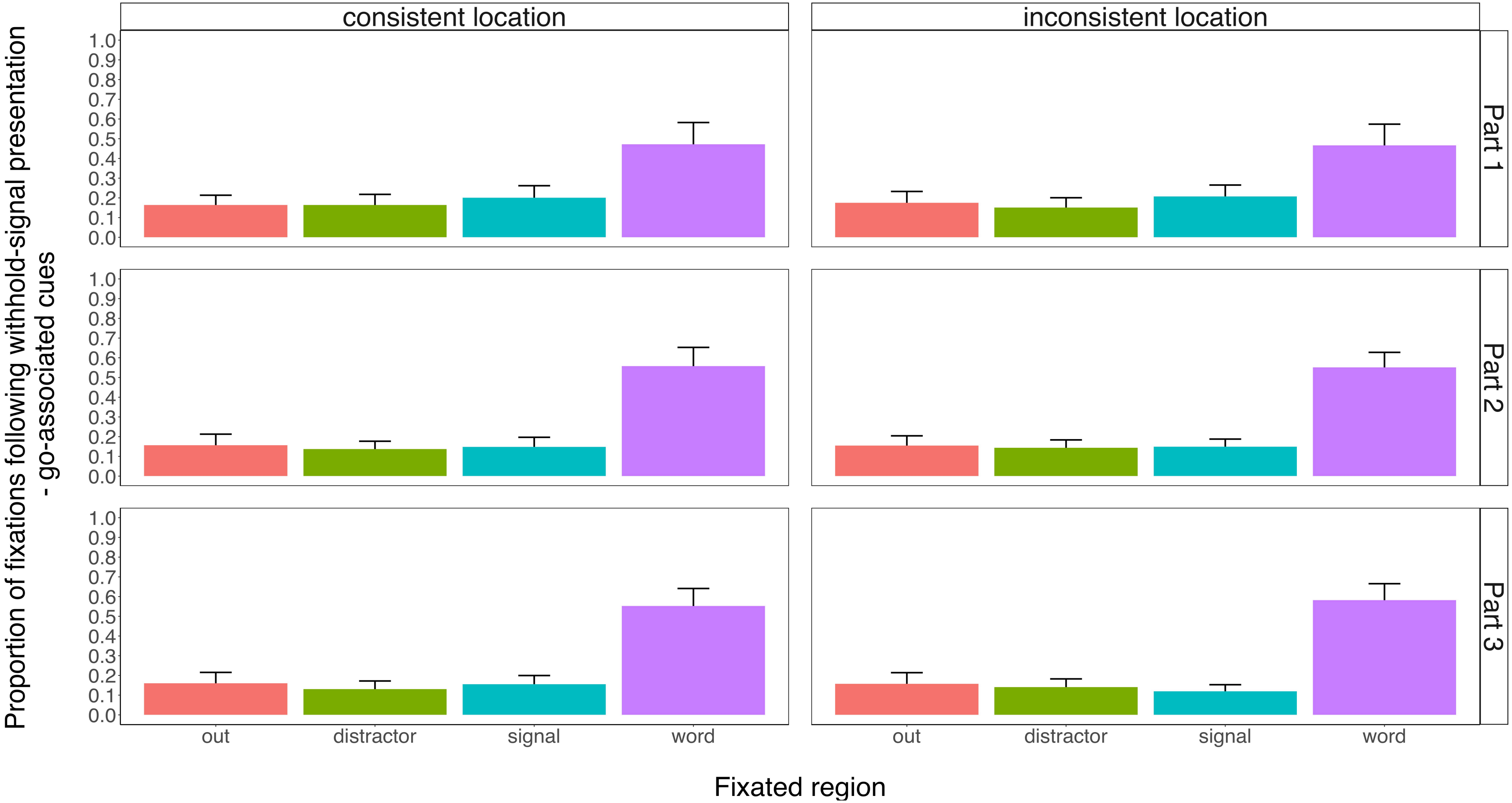

Figure S3: The proportion of fixations following withhold-signal presentation for go-associated cues as a function of region (word, signal, distractor, out), part (1-3), and signal location (consistent, inconsistent).

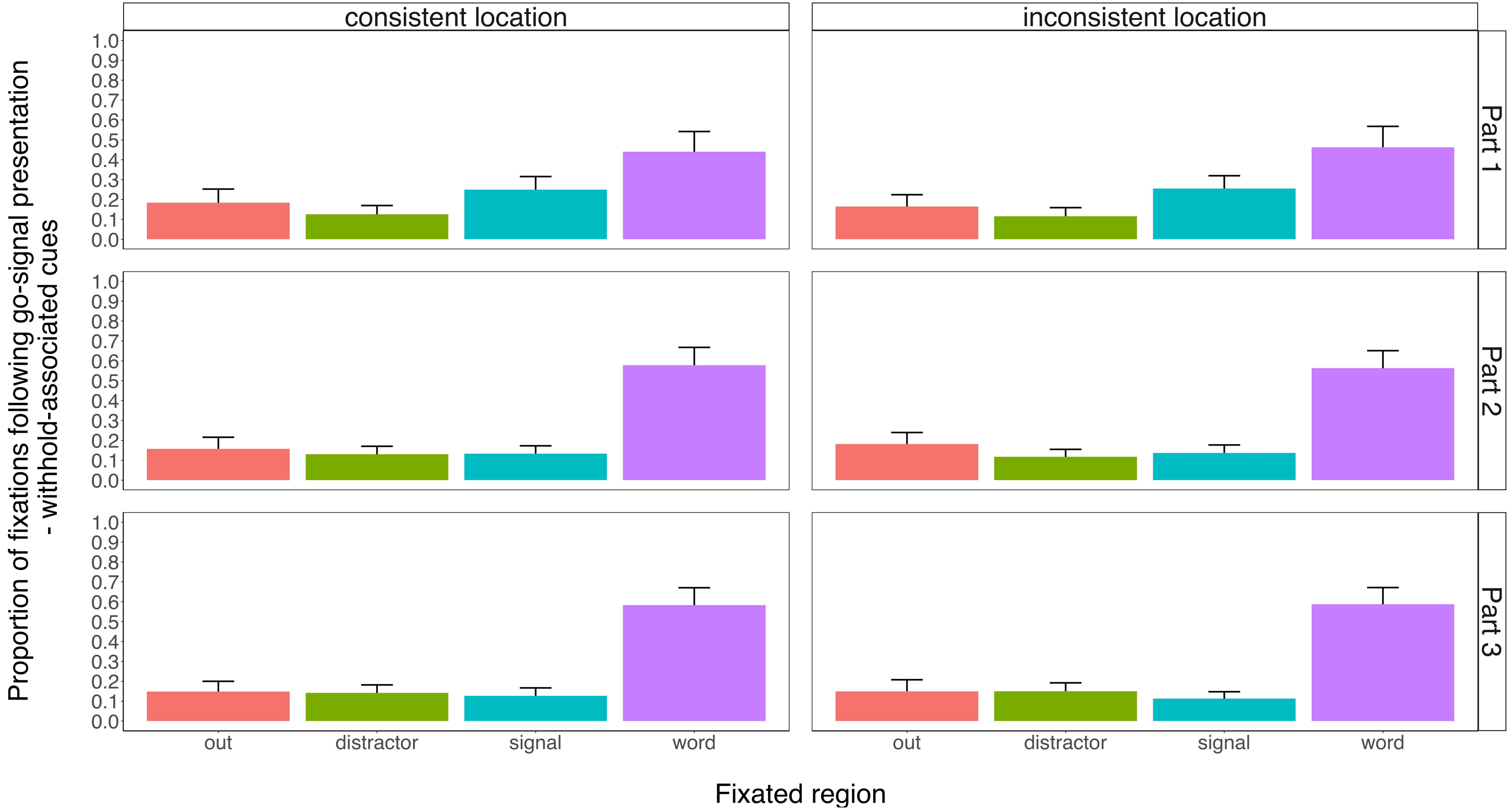

Figure S4: The proportion of fixations following go-signal presentation for withhold-associated cues as a function of region (word, signal, distractor, out), part (1-3), and signal location (consistent, inconsistent).

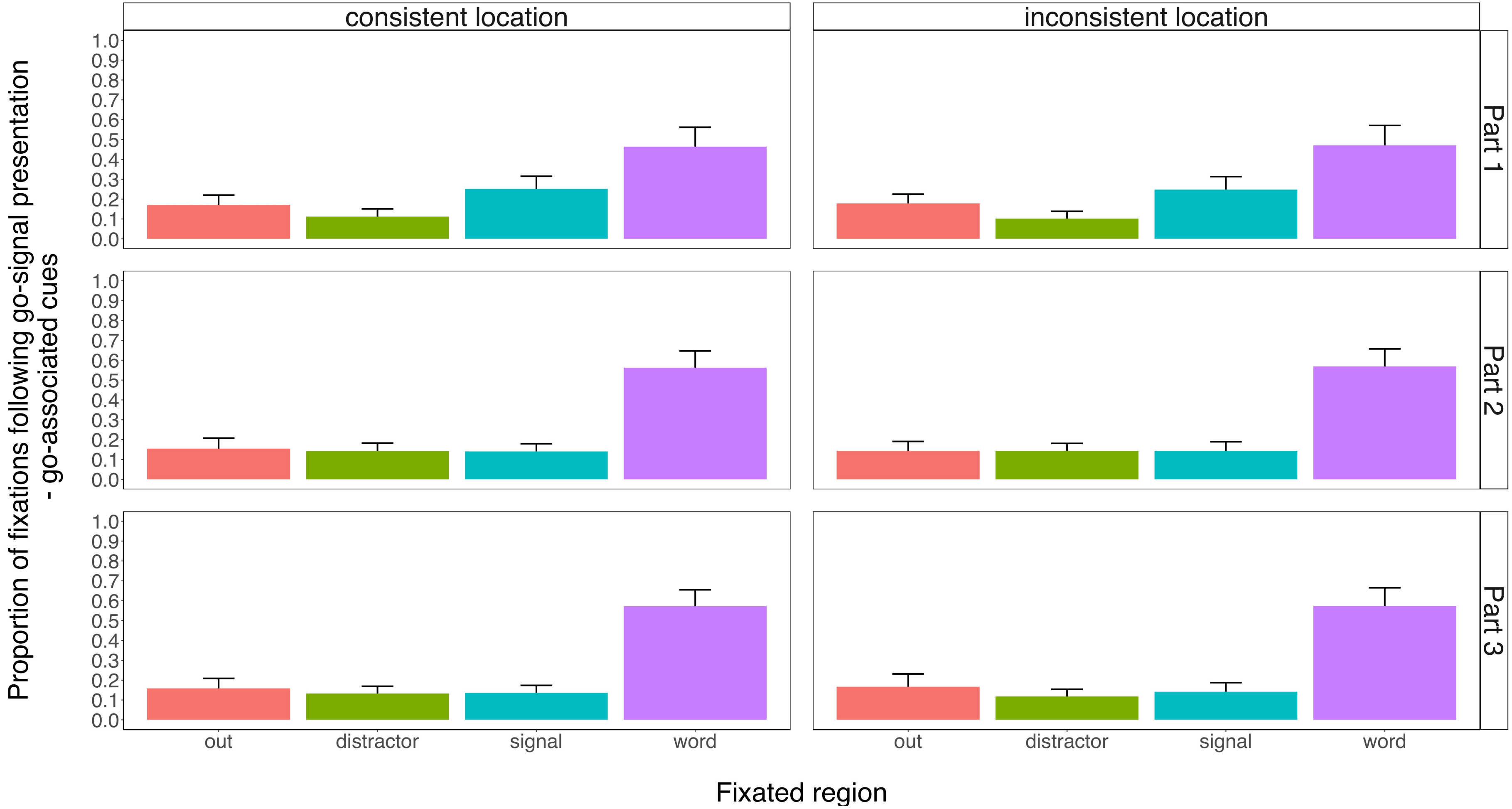

Figure S5: The proportion of fixations following go-signal presentation for go-associated cues as a function of region (word, signal, distractor, out), part (1-3), and signal location (consistent, inconsistent).

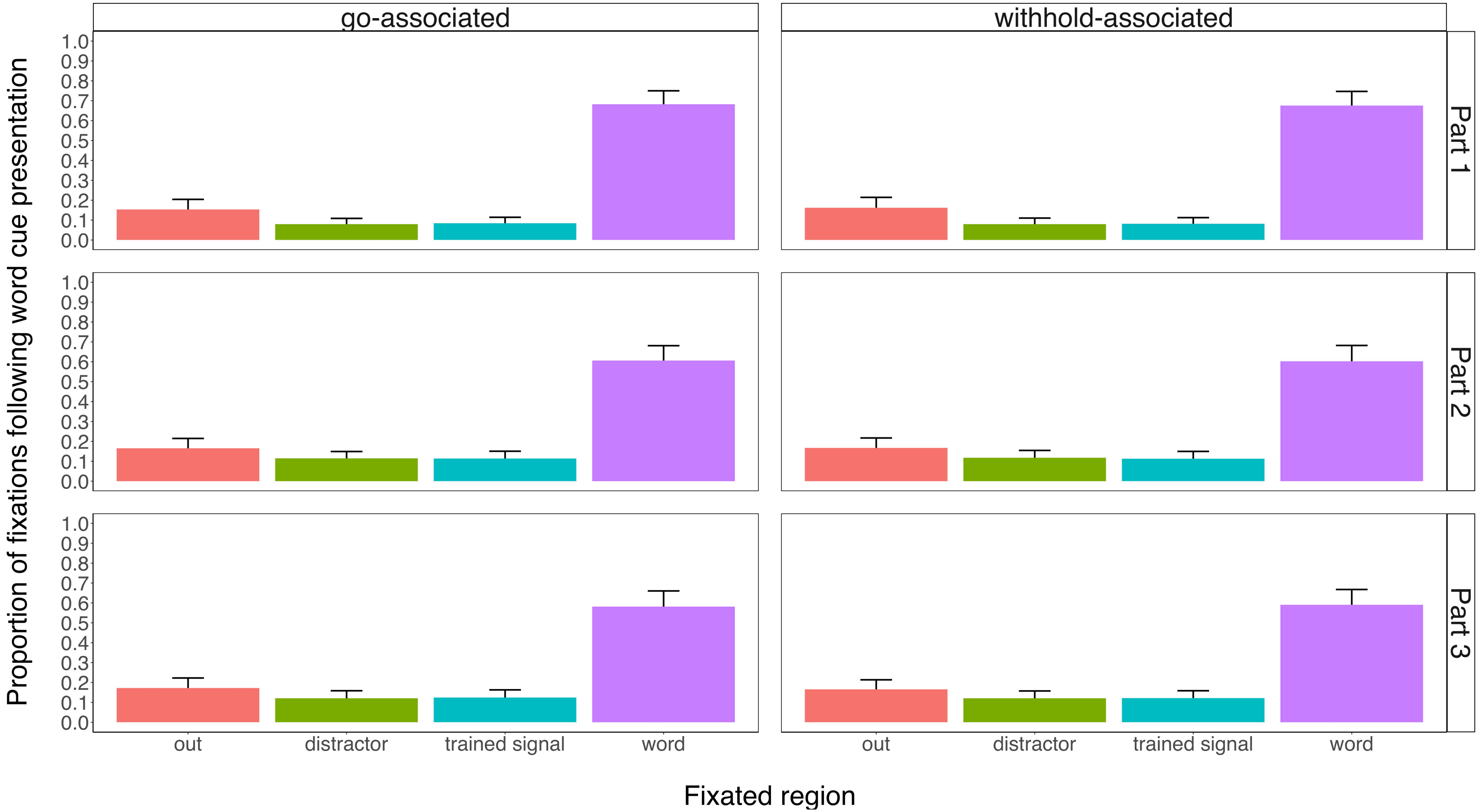

Figure S6: The proportion of fixations following word cue presentation as a function of region (word, trained signal, distractor, out), part (1-3), and go/withhold-type (go-associated, withhold-associated).

References

Brainard, D. H. (1997). The Psychophysics Toolbox. *Spatial Vision*, *10*(4), 433–436.

Brysbaert, M., & New, B. (2009). Moving beyond Kucera and Francis: a critical evaluation of current word frequency norms and the introduction of a new and improved word frequency measure for american english. *Behavior Research Methods*, 41,977–990. doi: 10.3758/BRM.41.4.977.

Cornelissen, F. W., Peters, E. M., & Palmer, J. (2002). The eyelink toolbox: Eye tracking with MATLAB and the psychophysics Toolbox. *Behavior Research Methods, Instruments, & Computers*, *34*(4), 613–617. doi:10.3758/BF03195489

Rehder, B., & Hoffman, A. B. (2005). Eyetracking and selective attention in category learning. *Cognitive Psychology*, *51*(1), 1–41. doi:10.1016/j.cogpsych.2004.11.001

R Development Core Team. (2014). R: A language and environment for statistical computing. Vienna, Austria: R Foundation for Statistical Computing. Retrieved from http://www.R-project.org/

Verbruggen, F., Stevens, T., & Chambers, C. (2014). Proactive and Reactive Stopping When Distracted: An Attentional Account. *Journal of Experimental Psychology. Human Perception and Performance*, *40*(4), 1295–1300.
